# Constraining the formation and transport of lunar impact glasses using the ages and chemical compositions of Chang’e-5 glass beads

**DOI:** 10.1126/sciadv.abq2542

**Published:** 2022-09-28

**Authors:** Tao Long, Yuqi Qian, Marc D. Norman, Katarina Miljkovic, Carolyn Crow, James W. Head, Xiaochao Che, Romain Tartèse, Nicolle Zellner, Xuefeng Yu, Shiwen Xie, Martin Whitehouse, Katherine H. Joy, Clive R. Neal, Joshua F. Snape, Guisheng Zhou, Shoujie Liu, Chun Yang, Zhiqing Yang, Chen Wang, Long Xiao, Dunyi Liu, Alexander Nemchin

**Affiliations:** ^1^ Beijing SHRIMP Center, Institute of Geology, Chinese Academy of Geological Sciences, Beijing 100037, China.; ^2^ Planetary Science Institute, School of Earth Sciences, China University of Geosciences, Wuhan 430074, China.; ^3^ Research School of Earth Sciences, The Australian National University, Canberra, ACT 2601 Australia.; ^4^ School of Earth and Planetary Sciences, Curtin University, GPO Box U1987, Perth, WA 6845, Australia.; ^5^ Department of Geological Sciences, University of Colorado Boulder, Boulder, CO 80309, USA.; ^6^ Department of Earth, Environmental, and Planetary Sciences, Brown University, Providence, RI 02912, USA.; ^7^ Department of Earth and Environmental Sciences, The University of Manchester, Manchester, M13 9PL, UK.; ^8^ Department of Physics, Albion College, Albion, MI 49224, USA.; ^9^ Shandong Institute of Geological Sciences, Jinan, Shandong 250013, China.; ^10^ Department of Geosciences, Swedish Museum of Natural History, SE-104 05 Stockholm, Sweden.; ^11^ Department of Civil and Environmental Engineering and Earth Sciences, University of Notre Dame, Notre Dame, IN 46556, USA.

## Abstract

Impact glasses found in lunar soils provide a possible window into the impact history of the inner solar system. However, their use for precise reconstruction of this history is limited by an incomplete understanding of the physical mechanisms responsible for their origin and distribution and possible relationships to local and regional geology. Here, we report U-Pb isotopic dates and chemical compositions of impact glasses from the Chang’e-5 soil and quantitative models of impact melt formation and ejection that account for the compositions of these glasses. The predominantly local provenance indicated by their compositions, which constrains transport distances to <~150 kilometers, and the age-frequency distribution are consistent with formation mainly in impact craters 1 to 5 kilometers in diameter. Based on geological mapping and impact cratering theory, we tentatively identify specific craters on the basaltic unit sampled by Chang’e-5 that may have produced these glasses and compare their ages with the impact record of the asteroid belt.

## Teaser

The chronology of Chang’e-5 impact glasses suggests a dynamical link between lunar impacts and collisions in the asteroid belt.

## INTRODUCTION

Particles of silicate glass formed either during volcanic eruptions or by impact melting are a ubiquitous component of all lunar soils. Commonly referred to as beads or spherules, they range in size from a few tens of micrometers to a few millimeters and typically have spherical, oval, or dumbbell shapes with broken fragments and shards also present. Glasses produced by impacts can be distinguished from those formed by volcanic eruptions using chemical and textural characteristics (*1*–*4*). Previous studies of the ^39^Ar-^40^Ar and U-Pb chronometric systems in lunar regolith glasses provide a foundation for investigating the volcanic and impact history of the Moon (*3*, *5*–*13*). Volcanic glasses provide unique information about the lunar mantle (*14*, *15*), whereas impact glasses reflect the compositions of crustal target materials (*16*–*18*) and collisional dynamics of the inner solar system (*4*, *9*, *19*, *20*).

This study focuses on impact glasses returned from the Moon by the Chang’e-5 mission (*21*). The Chang’e-5 landing site is located on a relatively young [~2 billion year (Ga) old] basaltic unit (*22*, *23*), designated as Em4 (the fourth oldest Eratosthenian-aged mare basalt in the region), at a distance of at least ~150 km from the nearest compositionally distinct highlands and mare basalt terranes (*24*, *25*). These characteristics place testable limits on the transport distance of ejecta from impacts that occurred within the Em4 unit versus impacts into different geologic units. Impacts within the Em4 unit should produce melts with chemical compositions consistent with that of the local regolith developed on the underlying mare basalt lava flows (*26*). Possible exceptions are volatile elements, such as alkali metals, which might be fractionated during the impact process. Exotic glasses produced by impacts outside of or beneath (>50 m) the Em4 unit (*25*) should be recognizable by their distinctive chemical compositions.

Complicating issues that inhibit a comprehensive interpretation of the chemical compositions and ages of lunar impact glasses include uncertainties over (i) the size distribution of impact craters represented by the regolith glasses, (ii) the range of glass compositions that can be produced in a single impact, and (iii) the significance of the age distribution of these glasses for lunar impact history (*13*, *20*, *27*). Here, we address some of these questions through quantitative impact modeling constrained by the compositions and formation ages of the Chang’e-5 glasses. A detailed comparison of the predicted dispersal of ejected melt with the size-frequency distribution of impact craters on the Em4 unit allows tentative identification of likely craters that contributed individual glass spherules to the Chang’e-5 regolith samples. We then briefly discuss possible implications for collisional events in the asteroid belt that may have generated the population of impactors responsible for formation of these craters.

## RESULTS

### Chemical compositions and chronology of Chang’e-5 glasses

**Fig. 1.**
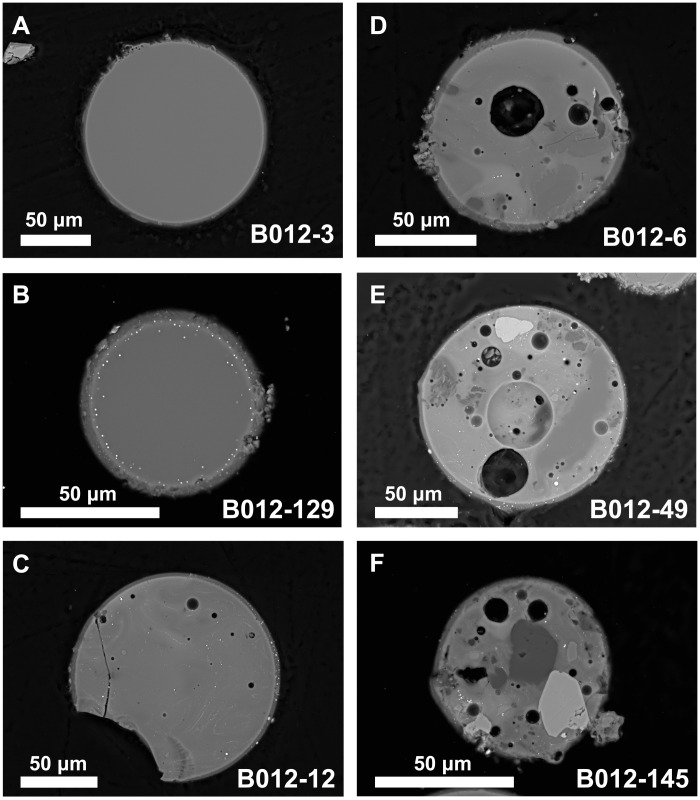
Textural range of Chang’e-5 glass beads. (**A**) Homogeneous (type 1a) glass with no clasts, schlieren, vesicles, or metal. Slight adhering regolith. (**B**) Sphere of homogeneous (type 1a) glass with metal around the rim. (**C** and **D**) Type 2 spheres with increasing proportion of schlieren, metal, and vesicles. (**E** and **F**) Type 3 spheres with partially digested clasts, schlieren, metal, and vesicles.

A set of 215 glass particles ranging in diameter from ~50 to 200 μm was selected on the basis of macroscopic characteristics such as smooth exterior surfaces and was classified on the basis of their textures and shapes (Fig. 1, fig. S1, and table S1). The textures range from homogeneous, vesicle-free glass to clast-rich particles containing up to 50% of unmelted mineral fragments and abundant vesicles. Flow banding in the glass and partly digested mineral fragments in a substantial fraction of the particles indicate incomplete melting and inhomogeneous melt (fig. S1). Glasses inferred to have been fully molten during their formation were classified as textural type 1 (fig. S1 and table S1), whereas beads with undigested clasts were classified as type 2 or type 3 depending on the abundance of clasts, proportion of vesicles, and shape (Fig. 1, fig. S1, and table S1). The general sense is a decreasing extent of melting and, therefore, peak time-temperature history going from type 1 to type 3 (Fig. 1). The presence of undigested mineral and lithic fragments in the textural type 2 and 3 melt particles demonstrates their formation by impacts.

**Fig. 2.**
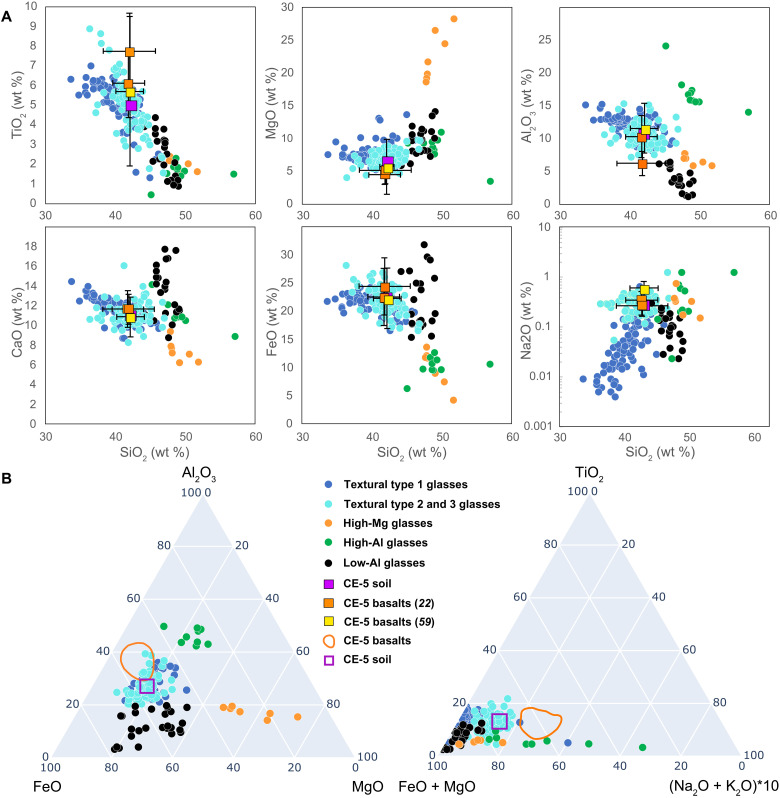
Major element variations in Chang’e-5 glass beads. (**A**) Major oxides versus SiO_2_ (wt %). (**B**) Ternary plots showing chemical compositions of the analyzed glass beads. Symbols in all plots: Blue circles, homogeneous glasses local to Chang’e-5 site (textural type 1); cyan circles, inhomogeneous glasses local to Chang’e-5 site (textural types 2 and 3); orange circles, high-Mg glasses; green circles, high-Al glasses; black circles, low-Al glasses; purple square, Chang’e-5 soil. Orange and yellow squares in (A) represent bulk compositions of Chang’e-5 basalts from Che *et al*. (*22*) and mean from Tian *et al*. (*59*), respectively (error bars represent 2σ errors). The orange field outlined in (B) represents the range of Chang’e-5 basalts.

The origin(s) of the fully molten type 1 glasses can be determined from their chemical and physical characteristics, following criteria described by previous studies (*1*–*4*), and ages of the glasses reported here. We interpret all of these particles as quenched impact melt. Major element compositions of about 80% of the glasses (*n* = 176), irrespective of their textural type, correspond closely to the Chang’e-5 bulk soil (*28*) and basalts (Fig. 2 and table S1) (*22*). On the basis of this correspondence, we categorized them as locally derived. Many of the fully molten beads from this chemical group are depleted in Na and K compared to the Chang’e-5 mare basalts and the bulk local soil (Fig. 2 and table S1), implying volatile loss during impact melting. The relatively low SiO_2_ contents of nearly half of the type 1 glasses [less than the 42 weight % (wt %) SiO_2_ determined for the bulk soil] also indicate volatile loss of SiO_2_, analogous to what has been observed in other suites of lunar impact glasses (*13*, *18*, *29*), and indicates temperatures well above the liquidus for the Em4 regolith composition (~1150°C).

A smaller, but still substantial, number of beads (*n* = 39) have chemical characteristics that distinguish them from the “local” group discussed above and are termed “exotic.” We classified the exotic particles into three chemical groups (Fig. 2): (i) high MgO > 18.7 wt % (*n* = 7), (ii) high Al_2_O_3_ > 14.1 wt % (*n* = 10), and (iii) low Al_2_O_3_ < 6.5 wt % (*n* = 22). These three exotic groups have higher SiO_2_ contents (45 to 56 wt %), compared to the local glasses, which mostly have <45 wt % SiO_2_ (Fig. 2). The high Al_2_O_3_ glasses also have higher Th concentrations between 9 and 25 μg/g compared to all other samples, where Th content does not exceed 7 μg/g and averages around 3 to 4 μg/g (table S1). The chemically exotic glasses have textures similar to the locally derived particles and are classified within the same three textural types. The apparent depletion of alkali metals and SiO_2_ in the locally derived type 1 glasses and the overall similarity of concentrations of more refractory elements in these glasses to those in Chang’e-5 soil and basalts imply that the fully molten particles with chemically local compositions were produced by impacts that occurred within the boundaries of the Em4 basaltic unit. Assuming that the Em4 unit excavated by these impacts is chemically homogeneous within the limits defined by the small number of currently available analyses of Chang’e-5 soil and basaltic fragments and chemically distinct from surrounding units (*25*), the exotic glasses must originate outside of the boundaries of Em4, implying transport distances in excess of 150 km or from depths > 50 m. Considering the relatively short transport distances estimated for pyroclastic deposits on the Moon (kilometers to tens of kilometers) (*30*), this supports an impact origin of exotic glasses of all textural types and chemical compositions. In addition, volcanic vents that can produce pyroclastic deposits are not found within the Em4 unit (*24*). Consequently, all investigated glass particles from our Chang’e-5 sample are interpreted as particles of impact melt, based on their textural and chemical characteristics. Additional support for an impact origin is provided by the ages of the glasses, with the majority being younger than the Chang’e-5 basalt unit Em4.

U-Th-Pb isotopic data were obtained on 166 glasses (table S1). All other particles either did not have sufficient Pb for an isotopic analysis [less than 1 count per second (cps) of ^206^Pb] or lacked a large enough area of homogeneous glass for analysis as determined by visual inspection of back-scattered electron images (fig. S1).

Critical for obtaining U-Th-Pb formation ages is an adequate correction for the amount and isotopic composition of Pb that did not form by in situ U-Th decay after formation of the beads. In principle, this extra Pb contribution could be a complex mixture of radiogenic decay products of U and Th formed on the Moon before and after the formation of a bead, such as (i) inherited Pb that was present in the target rock(s) at the time of impact and (ii) Pb that may have been introduced into the bead after it formed and during its residence in the lunar regolith (*13*). As Pb is a volatile metal, we assume that much of the Pb present in the target at the time of impact was lost, but this loss is not necessarily quantifiable. An added complication is that the Pb isotopic composition of the target is evolving due to U-Th decay, and this isotopic variability could contribute to both the pre-impact inherited and the post-impact introduced Pb in any given particle. Contamination from terrestrial Pb from a variety of potential sources also needs to be considered, as well as the addition of exogenous meteoritic Pb that is accumulated continuously in lunar soils. Assuming that nonlunar Pb in the glasses is dominated by the contribution from asteroid-derived materials and that terrestrial (laboratory) contamination was insignificant (see the Supplementary Materials for details), the formation ages of the beads and the isotopic composition of any unsupported lunar Pb (comprising both inherited and introduced components) can be estimated from individual analyses of the investigated glasses (table S1).

Textural and compositional characteristics of the studied glasses have implications for interpreting their U-Pb systems. The textures (described here as three different textural types) are a reflection of conditions of formation and/or transportation and deposition, which all can have an effect on the proportion of unsupported and introduced Pb. The compositions of the glasses help to distinguish beads that are produced by (i) impacts onto basaltic unit Em4 surrounding Chang’e-5 landing site and/or regolith developed above this unit from (ii) impacts outside the bounds of Em4 unit. The glasses formed in these two categories (referred to here as local and exotic, respectively) clearly come from different source terranes, and therefore were produced by discrete impacts, even when their ages are indistinguishable within uncertainties. Therefore, these local and exotic glasses must be discussed separately when interpreting the U-Pb data.

**Fig. 3.**
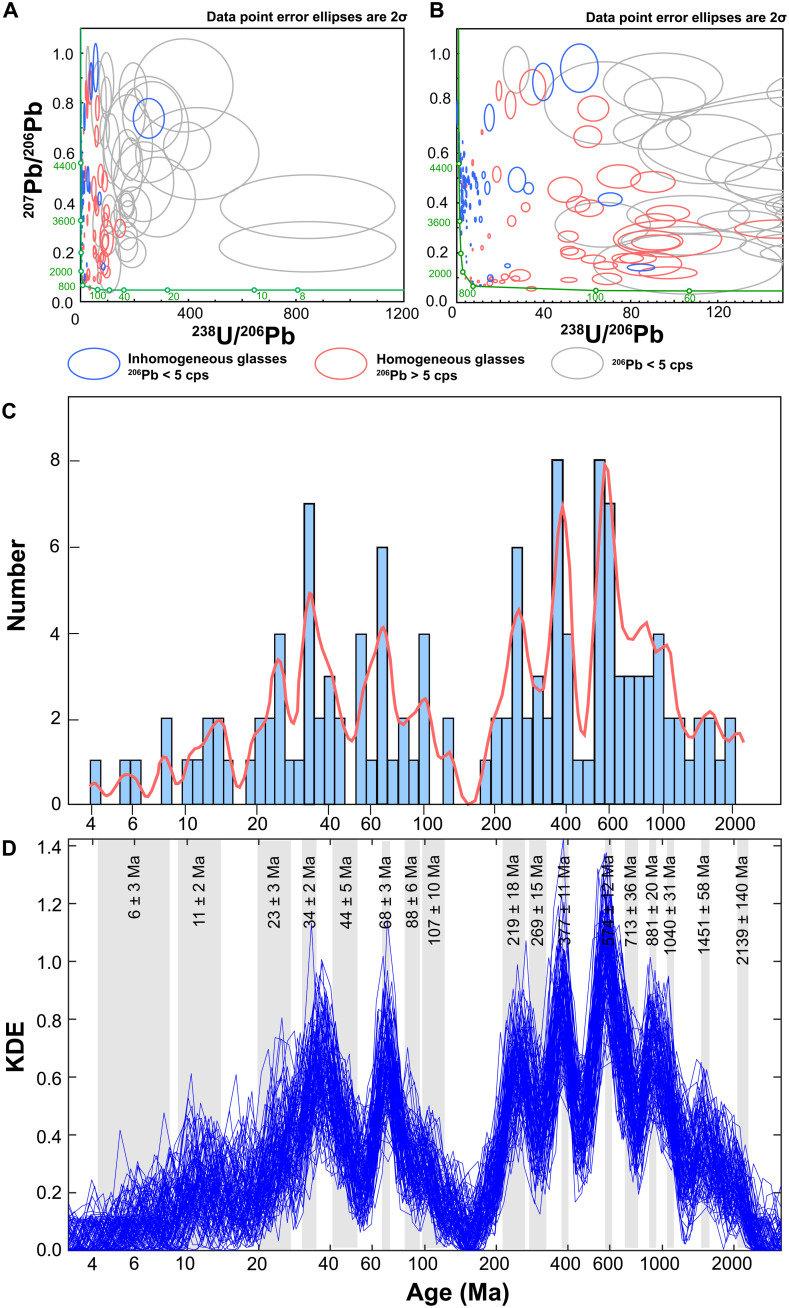
U-Pb isotopic compositions obtained for Chang’e-5 glass beads. (**A**) Tera-Wasserburg diagram showing measured isotopic compositions. Concordia is shown as green curve with the ages in million years shown next to it. Gray ellipses, analyses with ^206^Pb < 5 cps; red ellipses, homogeneous glasses (textural type 1) with ^206^Pb > 5 cps; blue ellipses, analyses of inhomogeneous glasses (textural types 2 and 3) with ^206^Pb > 5 cps. (**B**) Zoomed-in part of (A) showing analyses closer to vertical (older) part of the concordia curve. (**C**) Age distribution of the local compositional group of glasses of all textural types (kernel density: red curve and histogram). (**D**) Simulation of kernel density estimate (KDE) variance using 100 trials where dates are randomly selected for each of 135 local glass beads using their ages and uncertainties estimated from SIMS (Secondary Ion Mass Spectrometer) data (blue lines). Ages estimated for different groups using an unmixing approach (*32*) are shown as gray areas with their width corresponding to uncertainties estimated for each age. A logarithmic scale is used for the horizontal axis in (C) and (D) to emphasize individual groups younger than 100 Ma.

The clast-bearing and incompletely homogenized particles (types 2 and 3) tend to have uncorrected Pb isotopic compositions that fall close to the subvertical (older) part of the Terra-Wasserburg concordia curve, indicating greater proportions of older inherited Pb (Fig. 3, A and B). These heterogeneous glasses also account for most of the corrected ages >1 Ga, irrespective of the approach taken to correct the data (see the Supplementary Materials). In contrast, the fully molten beads (textural type 1) fall to the right of the concordia diagram at relatively young apparent ^238^U/^206^Pb ages [<2000 million years (Ma)] with some plotting near the younger, subhorizontal part of the concordia curve, suggesting a predominance of in situ radiogenic Pb.

After correction for unsupported Pb, we find that 135 impact beads of all textural types representing the local chemical group have formation ages that range from a few million years to 2 Ga old, the upper limit being that of Chang’e-5 basalt emplacement (table S1). These individual dates can, in principle, represent any number of separate impact events between the maximum of 135 and the minimum number of statistical groups defined by the distribution and uncertainties of all individual dates. Untangling information contained in such a dataset obtained from a population of lunar impact glasses presents a similar challenge to that faced by studies of detrital zircon populations in terrestrial sedimentary rocks, where individual zircon grains may represent separate source rocks while groups of individual grains similar in age could come from the same source. Therefore, methods developed to visualize detrital zircon data, which include plotting data as histograms, probability density plots, and kernel density plots to identify existing peaks in the populations (*31*), can be similarly applied to studies of lunar glasses. In addition, statistical techniques, such as “unmixing” (*32*) developed to define possible statistically homogeneous age groups in a zircon population can help to determine the minimum number of impacts required to produce an observed age distribution in a population of lunar impact glasses. While not perfect, a combination of these techniques is the best available approach for a study of both detrital zircon populations and lunar impact glasses. Histogram and kernel density plot constrained for Chang’e-5 glass beads (Fig. 3C) indicate a number of peaks and troughs suggesting either a relatively small number of impacts responsible for the formation of the beads population at the landing site or substantial variation of impact rate through time. The former assumes that the glasses with ages indistinguishable within their uncertainties originate in the same impact, while the second interpretation allows for a possibility of beads with similar ages to form in multiple impacts with age differences that cannot be resolved within currently achievable analytical uncertainties. Applying an “unmixing” approach (*32*) to the Chang’e-5 glasses identifies 17 statistically significant groups (Fig. 3D and table S2), ranging from 6 ± 3 Ma to 2139 ± 140 Ma (all uncertainties at the 95% confidence level), where individual dates within each group are indistinguishable within uncertainties. The glasses with Pb concentrations below detection could be even younger, but it is not possible to define specific age groups for these samples. Variance of the age distribution pattern shown in Fig. 3C can be tested by a simulation in which random formation ages for the glasses are drawn from the measured dates of the 135 beads and their uncertainties, assuming a normal distribution around the mean dates. Repeating this process multiple times and plotting a kernel density distribution for each randomly derived set (Fig. 3D) show the level of variance in the age distribution pattern defined by the combined uncertainties of individual dates. This simulation (Fig. 3D) indicates that larger peaks in the distribution defined by more than eight individual glass beads (table S2) remain clearly identified even when individual uncertainties are taken into account. Peaks determined by the smaller number of glasses are less obvious, although some are still visible (Fig. 3D). Nevertheless, these latter age groups are still significant even if their ages are defined with a lower degree of accuracy, as the glasses in them cannot be statistically assigned to the more prominent groups.

The U and Th concentrations in the fully molten, type 1 local glasses span broad ranges (U = 0.2 to 2.2 μg/g; Th = 0.7 to 6.3 μg/g) with average abundances marginally lower than that of the bulk Chang’e-5 soil (*28*) (U = 1.0 versus 1.4 μg/g; Th = 2.9 versus 4.7 μg/g). Analyzed spots in the texturally heterogeneous beads (types 2 and 3) span larger ranges in U and Th contents (up to 7 and 14 μg/g for U and Th, respectively) but these may not represent the bulk composition of the entire beads, and the average U and Th abundances of these heterogeneous beads are similar to those of type 1.

The compositionally exotic glasses (high-Al, low-Al, and high-Mg groups) also show a range of formation ages between 8 ± 3 Ma and 1425 ± 120 Ma, with each compositional type containing multiple age groups of glasses. Although the nominal dates of four particles exceed the age of Em4 unit, they are within uncertainty of the basalt crystallization ages. The range of dates in the exotic glasses implies multiple impacts into their source regions, but the small number of beads in each of these compositional groups precludes precise age assignments.

## DISCUSSION

### Numerical modeling of impact ejecta

There is currently no general model for quantifying the production and spatial distribution of lunar impact glasses that would underpin a better understanding of their broader geological significance. Here, we present the results of numerical impact simulations that produce melt in small craters and discuss their implications for understanding the geology around the Chang’e-5 landing site and the provenance of lunar impact glass beads.

Particles of impact melt can form via nucleation in ejecta vapor and/or melting of the ejected material during jetting and possibly blanket formation. Theoretical modeling suggests that vapor nucleation would form spherules with a maximum estimated size of a few micrometers for a crater diameter of 1 km and an impactor velocity of 27 km/s and submicrometer sizes for smaller craters and more extreme velocities (*33*, *34*). Most spherules predicted to form by vapor nucleation are at least an order of magnitude smaller than the Chang’e-5 particles studied here. We, therefore, investigated ejecta melting as the mechanism responsible for formation of lunar impact glasses (*20*, *33*, *34*).

Numerical impact simulations were made using the iSALE-2D shock physics code (https://isale-code.github.io/) to investigate ejecta formation in craters between 100 and 1300 m in diameter, reflecting the constraint that only a few craters identified within the Em4 unit exceed 1 km in diameter. Numerical impact simulations investigating spherule-forming conditions focused on the early cratering phase in high resolution, which allowed estimates of the maximum landing distance and investigation of the early ejecta subject to the highest shock pressures and temperatures. Ejecta temperatures considered appropriate for spherule formation were constrained to be between 1100 and 2000 K (827° to 1727°C). This range was chosen on the basis of estimated solidus and liquidus temperature (~900° to 1150°C) for materials compositionally similar to Chang’e-5 regolith and basalts and conditions at which volatile loss of Si from the melts becomes recognizable in the samples (~1500°C) (*35*, *36*). Only the fraction of ejecta that was moving slower than the escape velocity of the Moon (~2.4 km/s) was considered.

Details on the numerical calculations can be found in the Supplementary Materials. Three of the models simulated collisions leading to craters with diameters of 100 to 620 m, calculated assuming that the target consisted of a 7-m-thick regolith layer over bedrock (table S3), which is appropriate for the area surrounding the landing site (*37*). Two-layer models were adopted for these smaller craters to account for the effect of different target cohesion and porosity between layers on the distribution of ejecta material (ejecta speed, ejecta angle, and deposition distance). Impact craters with larger diameters in the investigated crater size range (830 to 1300 m in diameter) imply excavation depths of 100 to 200 m (table S3), reducing the influence of regolith on the impact ejecta distribution. Consequently, consolidated rock was assumed as a target for the impact simulations for larger crater sizes.

**Fig. 4.**
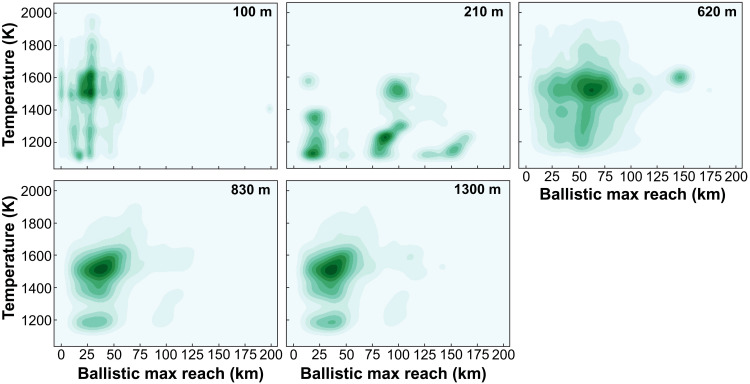
Maximum ballistic reach versus temperature for the early ejecta melt in the five crater sizes. The plots show kernel density estimates, which is a mathematical interpretation representing the statistical likelihood for a parameter value. The data were extracted out of the numerical impact simulations for postprocessing.

**Fig. 5.**
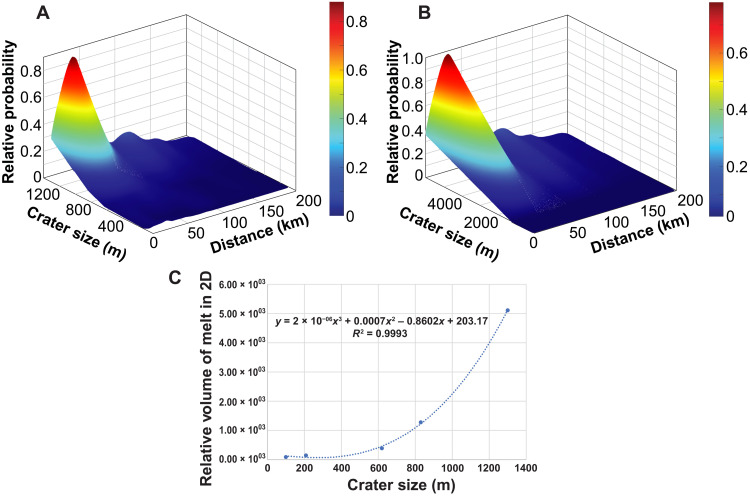
Probability of finding glasses from specific impact craters related to distance of transportation to the landing site and crater diameter (colors reflect changing probability). (**A**) Probability estimates confined to six model runs (100 to 1300 m in diameter). (**B**) Probability estimates extrapolated to the craters up to 6 km in diameter. (**C**) Relationships between crater size and relative volume of the melt produced for six model runs (100 to 1300 m in diameter). The relative volume of melt in two dimensions (2D) refers to a cumulative number of numerical cells in an impact simulation that satisfied the spherule-forming conditions multiplied by the physical area the numerical cells represent. This is an indicator for volume but only calculated for a vertical crater cross section.

The numerical impact simulations (summarized in Fig. 4 and table S3) suggest that impacts forming craters between 100 and 1300 m in diameter all produce ejected material that meets the melting criteria (fig. S5; see the Supplementary Materials for details), with the volume of spherule-forming melt increasing with the crater volume (Fig. 5C), consistent with previous observations (*20*, *33*, *34*). Estimated ejecta temperatures can reach between 1100 and 1700 K (827° to 1427°C), suitable for producing the partially and fully molten beads based on calculated melting temperatures of the local regolith, with a fraction of the ejected material reaching temperatures exceeding 1900 K (1627°C), suitable for volatile loss of Si, in the larger craters. These temperature estimates were made using post-shock temperatures, tracked with high numerical confidence. Considering that the spherule-forming material was captured during the very early crater excavation phase, the ejecta affected by the shock and post-shock temperatures remains comparable. The mean velocity of ejected material meeting the spherule-forming conditions is, on average, 300 to 500 m/s in all craters, resulting in transportation distances up to several tens of kilometers, assuming a flat surface and an assumption of a ballistic trajectory as well as no friction in the ejecta. Similar transportation distances estimated for craters of all sizes are the consequence of a consistent set of assumptions about impactor velocity, target conditions, and temperature limits where melting can occur. Impact simulation for a 200-m crater diameter produced more variability in ejecta temperature and speed compared to other crater sizes (Fig. 4). The likely explanation is that the estimated excavation depth for a crater of this size is 15 m (table S3), which provides a subequal sampling of the two layers (regolith and bedrock) for the assumed regolith thickness of 7 m. This indicates that the regolith composition and local structure can have substantial influence on ejecta behavior, and the set of model parameters explored here may not capture the full range of conditions in lunar impacts. Target and impact conditions (impact angle, size, and speed) would have a moderate effect on the ejection process (*38*, *39*); therefore, here, we limit the investigation to a combination of impact and target variations suitable for lunar regolith and small impacts on the Moon. Consequently, these models provide a baseline scenario in which the volume of melt produced in each crater and landing distance of ejecta would be the main factors that define the probability of glass from individual impact craters occurring in the Chang’e-5 soil sample (Fig. 5).

### Relationships to local geology

Here, we integrate the ejecta simulations into a geological model that predicts the probability of finding glasses from specific impact craters (i.e., volume of glass from a crater that can reach the landing site relative to the total volume of glass from all craters identified within the Em4 unit) as a function of their size and distance to the Chang’e-5 landing site (Fig. 5). We then compare these predictions with the observed crater size-frequency distribution (CSFD) within the Em4 unit to identify potential source craters that may have contributed impact glasses to the soil sample collected by Chang’e-5.

**Fig. 6.**
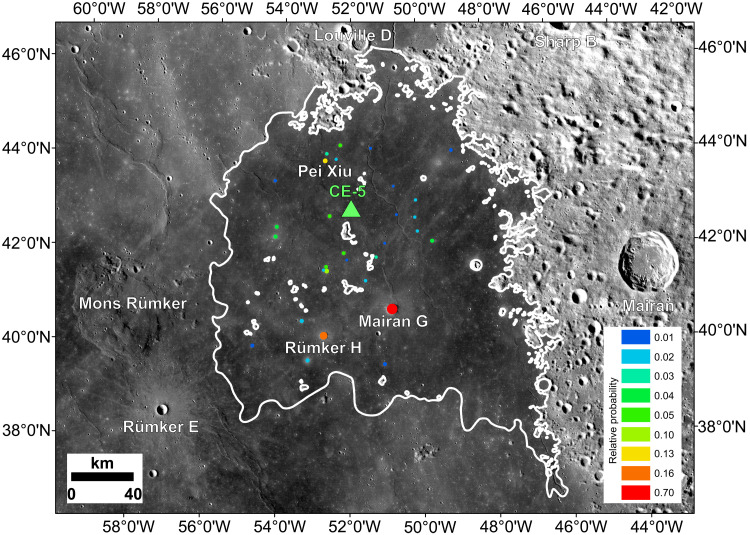
Geographical distribution of potential source craters that may have contributed impact melt to the Chang’e-5 landing site. The location of craters relative to Chang’e-5 landing site and estimated probability of these craters to be represented in Chang’e-5 sample glass beads population is shown by different colors. The white lines indicate the boundary of Em4, and the small white patches within Em4 are scattered kīpukas. The green triangle represents the landing site, the size of the circles is proportional to the size of the craters, and the color of the circles reflects the relative probability of source craters for the impact beads studied here.

The increase in melt volume with increasing crater size predicted by modeling and the high temperatures experienced by many of the local group glasses (as shown by their depletion of Si and alkalis) favor the formation of these glasses in larger (kilometers in diameter) craters. The models also suggest that most of the molten ejecta would be deposited within 100 km from a crater with a maximum deposition at around 20 to 50 km. From these constraints, a probabilistic model that relates the presence of molten ejecta in the soil to crater size and distance to the landing site can be applied to the 122,021 individual primary impact craters larger than 100 m that have been identified within the Em4 unit (table S4). Assuming that the number of regolith glass particles formed in an impact is proportional to the fraction of molten ejecta, the probability of finding impact glasses in Chang’e-5 soils originating from the three largest craters (diameters, 2.2 to 5.9 km) is >10%, whereas 15 additional craters with diameters of ~1.2 to 2.1 km returned probability estimates of >2%, and an additional 12 craters with diameters of ~1.0 to 1.8 km have probabilities of >1% (Fig. 6 and table S4). These 30 craters include the largest craters that have been identified within the Em4 unit (only 66 craters are ≥1 km), and they all occur within 110 km from the landing site (table S4). In this analysis, the vast majority of the >120,000 craters in the Em4 unit returned less than a 0.1% probability to have contributed to the Chang’e-5 impact bead population (Fig. 6), mostly due to their small size and resulting small estimated melt volumes (99.6% of the cataloged craters on the Em4 unit are <500 m in diameter). This suggests that impact craters of this size did not contribute substantially to the impact glass population at the Chang’e-5 landing site. These small craters produce small overall melt volumes, and only a trivial fraction of melt is sufficiently hot to fully melt the target or volatilize Si.

Comparing the CSFD model ages of 30 craters with an estimated relative probability of >1% for contributing glasses to the Chang’e-5 soil sample (table S4) with the age groups determined from U-Pb analysis of glasses in the studied Chang’e-5 sample provides an independent way to evaluate the consistency and accuracy of the geological and geochemical cratering ages. The largest crater in the Em4 unit (Mairan G, ~6 km in diameter) has the highest estimated relative probability. This crater has a CSFD model age of 479 ± 100 Ma (2σ), which is within uncertainties of both the 574 ± 12 Ma and 377 ± 11 Ma U-Pb age groups that are defined by the largest and second largest numbers of glasses (16 and 14, respectively) (table S2). Taking existing uncertainties into account, this crater can be viewed as the source of glasses for either of these U-Pb groups, or a combination of some glasses from both groups.

In contrast, 14 of the 30 craters have CSFD model ages between 53 ± 12 Ma and 73 ± 10 Ma, including the second largest crater, Rümker H (table S4). These CSFD model ages may be aligned with the U-Pb age group at 68 ± 3 Ma (*n* = 12; table S2), possibly suggesting that several of these craters contributed the glasses that define this U-Pb age group.

While the apparent agreement of some of the significant CSFD model ages of large craters in the Em4 unit and U-Pb age groups is encouraging, it is evident that the 30 craters with a high (>1%) probability of delivering impact glasses to the Chang’e-5 landing site (table S4) can explain only about half of the age groups defined from U-Pb analyses of glass beads (table S2), with the younger and older U-Pb age groups remaining unaccounted for. Therefore, some of the youngest and oldest U-Pb age groups may require cumulative contributions from multiple, low-probability craters with similar ages.

In summary, investigation of the chemistry and U-Pb ages of impact glasses in the studied Chang’e-5 sample, combined with modeling of the distribution of ejected glasses and CSFD model ages of selected craters, indicates that the 17 statistically defined U-Pb age groups of “locally” derived glasses represent the minimum number of impacts needed to produce the observed age populations of the glass beads. The true number of impacts contributing to this set of glasses is likely to be larger, although the actual number is difficult to constrain from the data and models currently available.

Previous studies have demonstrated the predominantly local provenance and relatively young ages of the majority of lunar impact glasses at a given site and their formation in modest size events have been suggested (*3*, *5*, *10*, *27*, *40*). However, neither the precision on the isotopic ages nor the constraints on local geology and CSFD model ages of potential parent craters have been adequately defined to allow a detailed comparison in those previous studies. Here, we have shown that combining chemical and chronological data for Chang’e-5 impact glasses with impact ejecta modeling helps to quantify some of these previous inferences, limiting the distance of transport of the locally derived glasses to about 100 km and suggesting kilometer-sized craters as sources of the majority of impact glasses in lunar soils. The small number of glasses identified as exotic to the landing site based on their chemical compositions (*n* = 26) makes it difficult to evaluate their age and crater size distributions relative to the locally derived glasses. Nevertheless, together with previous observations (*17*, *40*), they support the hypothesis that small populations of glasses in lunar soils were transported over much greater distances (in excess of 150 km) although the impact conditions necessary to produce such populations remain poorly defined.

### Glass beads and solar system dynamics

Age populations of impact glasses in the Chang’e-5 soil defined by U-Pb isotopic compositions, combined with CSFD model ages of individual craters and model probabilities of glass delivery from different craters to the landing site, suggest discrete episodes of impact cratering that varied over relatively short time scales. If this is correct, it implies that the age-frequency distributions of lunar impacts might provide information about dynamical events and processes that deliver impactors to the inner solar system (*41*, *42*).

Cosmic ray exposure (CRE) ages of asteroid-derived meteorites imply a relatively small number of collisions ejected them from their parent bodies (*43*, *44*). For example, CRE age clusters at 6, 13, 25, and 36 Ma in HED (Howardite-Eucrite-Diogenite) meteorites probably reflect impact events on their parent bodies at those times (*41*, *45*). U-Pb age populations of the Chang’e-5 glasses at 6 ± 1 Ma, 11 ± 2 Ma, 23 ± 3 Ma, and 34 ± 2 Ma are remarkably similar to these HED ejection ages. Although the ~1- to 5-km-diameter craters that are likely sources for the locally derived Chang’e-5 impact glasses would imply impactors larger than most meteorites (10 to 100 m) (*46*), substantial craters with similar ages are found on Earth [e.g., Karla: 10 km, 5 ± 1 Ma; Shunak: 3 km, 12 ± 5 Ma; Ries: 24 km, 14.81 ± 0.01 Ma; Haughton: 24 km, 23.4 ± 1 Ma; data from (*47*)]. This raises the possibility that a population of larger impactors accompanied breakup of the meteorite parent bodies, and these populations are recorded in impact events on Earth and the Moon.

The U-Pb age groups at 68 ± 3 Ma and 34 ± 2 Ma are notable because they coincide precisely with the ages of the terrestrial Chicxulub impact crater, a cluster of Late Eocene craters (including the 100-km-diameter terrestrial crater Popigai), respectively, and impact age peaks in HED and some ordinary chondrite meteorites. Although this may be coincidental, 14 of the 30 lunar impact craters studied here have CSFD model ages between 53 ± 12 Ma and 73 ± 10 Ma, including the second largest crater, Rümker H (IC-22) (table S4), which could be aligned with the 68 ± 3 Ma U-Pb age group. The 10-km impactor that created Chicxulub is proposed to originate in the outer asteroid belt through accumulation of weak orbital resonances (*48*–*50*). Such a process might not be expected to produce a large number of smaller accompanying impactors, although several substantial craters on Earth do have well-established ages within ~10 Ma of Chicxulub [e.g., Boltysh, Kara, Manson, and Lappajärvi (*51*)]. Therefore, a cluster of lunar craters at this time would not be unreasonable. In addition, at least four large terrestrial craters have isotopic ages of 35 to 38 Ma (Popigai, Chesapeake, Wanapitei, and Mistastin), coincident with a spike in extraterrestrial He and enrichments in meteoritic chromite found in sediments worldwide (*47*, *51*–*53*). These terrestrial Eocene crater-forming impactors appear to have been various types of ordinary chondrites (*47*, *53*), whereas the ^3^He may be coming from comets or interplanetary dust (*52*). In any case, this suggests an episode of perturbations that delivered impactors to the inner solar system, in which case an enhanced flux to the Moon at this time would be expected.

Last, thermal resetting ages of L-chondrite meteorites and their delivery to Earth at 460 to 480 Ma document a major collision in the asteroid belt (*54*–*56*), which produced a spike in the number of Ordovician age terrestrial craters (*47*). The largest crater in the Em4 unit (Mairan G, ~6 km in diameter) has a CSFD model age of 479 ± 100 Ma, identical to the age inferred for the L-chondrite parent body breakup event, and two of the most abundant U-Pb age peaks of the lunar glasses occur at 377 ± 11 Ma and 577 ± 12 Ma. Although the impactors represented by these two U-Pb age groups may not have been produced directly by the L-chondrite event, several asteroid families have either cratering or fragmentation ages between ~300 and 600Ma (*57*, *58*), possibly providing an ample supply of modest sized impactors that would not necessarily be seen in the terrestrial cratering record (*45*, *51*).

The data obtained from impact glasses in the Chang’e-5 soil sample and from regional studies of craters within the Em4 unit give an initial indication that lunar glass populations may provide a proxy for short-term variations in impact rates and possible relationships to dynamical processes in the asteroid belt. Cross-site comparisons with other lunar soils and regional crater ages will be necessary to distinguish Moon-wide events of particular significance from the background impactor flux.

## MATERIALS AND METHODS

Previous studies of impact glasses (*1*, *7*, *8*, *13*, *27*, *40*) identified a set of physical aspects that have impeded a deeper understanding of the chemistry and ages of impact glasses in the lunar regolith. These aspects include, but are not limited to, typical sizes of craters responsible for the major contribution of glasses to lunar soils, limits on transport distances, and influence of regolith thickness and the nature of underlying bedrock on impact melt production on the Moon. The aim of an improved understanding of the factors that influence the distribution of impact glasses in lunar soils played a major role in the initial design of this study. Therefore, a multidisciplinary study that combined chemical and chronological studies of samples, modeling of impact ejecta distribution, and integration of geological data obtained by remote sensing studies was applied to constrain the physical and chemical processes that help to form glass populations of the studied samples. This multidisciplinary approach helps link individual samples to the broader regional geology, potentially helping to identify target regions and even individual craters on the Moon responsible for populations of impact glasses at this and other landing sites. The Chang’e-5 landing site has a relatively simple geology compared to other sites where samples are available for laboratory studies. It is located in the middle of a vast and relatively young (~2 Ga) basalt unit with an estimated thickness of ~50 m, with nearly 150 km to the boundaries of the unit with a relatively constant thickness of regolith. Hence, the sample provides an opportunity to define the significance of lunar impact glasses in determining processes responsible for their formation, better than has been possible previously.

Two hundred fifteen glass beads of >50 μm in diameter (fig. S1) were selected from 2 g of Chang’e-5 lunar regolith (CE5C0400YJFM00402) allocated by the China National Space Administration (CNSA). They were mounted in epoxy resin to make four 25-mm-diameter mounts (B012, B013, B014, and B017), together with two terrestrial synthetic basalt glass standards (USGS BCR-2G and BHVO-2G) and a synthetic glass standard (NIST SRM 610). The analysis of the glasses followed sequence of techniques developed and tested in previous studies:

1) Initial petrographic characterization of all 215 glasses has been made using the ZEISS MERLIN Compact Scanning Electron Microscope at the Beijing SHRIMP Center, Institute of Geology, Chinese Academy of Geological Sciences, Beijing.

2) Chemical compositions of homogeneous areas of 202 individual glasses were obtained using the JEOL JXA-8100 electron microprobe at Institute of Geology, Chinese Academy of Geological Sciences, Beijing and the JEOL JXA-8230 electron microprobe at Shandong Institute of Geological Sciences, Jinan. Duplicate analyses have been made in 5 larger glasses, while 13 glass beads belonging to textural type 3 with abundant fragments and vesicles were excluded as they do not have homogeneous glass areas large enough to place electron microprobe spot.

3) U-Th-Pb data were collected for 166 glasses using a SHRIMP IIe MC ion microprobe at the Beijing SHRIMP Center, Institute of Geology, Chinese Academy of Geological Sciences, Beijing. The remaining glasses did not contain sufficient Pb to yield reliable U-Pb SHRIMP analysis.

4) After SHRIMP analysis, all U-Pb dating pits were inspected by ZEISS MERLIN FESEM to confirm the position of the analyses and to inspect for the possible unseen inclusions or cracks in the samples.

These data restricted input/output parameters for the modeling of impact ejecta and geological interpretations. The geochemical data limited the sizes of craters to those present within the basaltic unit sampled by the landing site and the temperatures of the melt particles required to satisfy the range of chemical compositions and textures of investigated melt. Modeling results have further defined the relationships between crater size, transport distance, and statistical probability of finding impact melt droplets in the soil sample. This statistical probability is combined with the spatial distribution of primary craters larger than 100 m within the boundaries of the Em4 basalt unit obtained from remote observations helped to identify craters with the highest likelihood of being represented in Chang’e-5 sample. Detailed description of analytical protocols, analytical data reduction, modeling, and probability estimation is provided in the Supplementary Materials.

## Supplementary Materials

### This PDF file includes:

Supplementary Text Figs. S1 to S6 References

## Notes

View/request a protocol for this paper from *Bio-protocol*.
