# Supplementary material for: Constraining the formation and transport of lunar impact glasses using the ages and chemical compositions of Chang’e-5 glass beads
Source: Sci Adv. 2022 Sep 28;8(39):eabq2542. doi: 10.1126/sciadv.abq2542 (PMC9519047; doi:10.1126/sciadv.abq2542)
Supplement: Supplementary file 1 — Supplementary Text Figs. S1 to S6 References [file sciadv.abq2542_sm.pdf]

Science Advances
AAAS

Supplementary Materials for

Constraining the formation and transport of lunar impact glasses using the ages and chemical compositions of Chang'e-5 glass beads

Tao Long *et al.*

Corresponding author: Tao Long, longtao@bjshrimp.cn

Sci. Adv. **8**, eabq2542 (2022)
DOI: 10.1126/sciadv.abq2542

The PDF file includes:

Supplementary Text
Figs. S1 to S6
Legends for tables S1 to S4
References

Other Supplementary Material for this manuscript includes the following:

Tables S1 to S4

Supplementary Text

Materials and Analytical Methods

Sample Preparation

215 glass beads of >50 μm diameter (Fig. S1) were selected from 2 g of CE-5 lunar regolith (CE5C0400YJFM00402) allocated by the China National Space Administration (CNSA). They were mounted in epoxy resin to make four 25 mm diameter mounts (B012, B013, B014 and B017), together with two terrestrial synthetic basalt glass standards (USGS BCR-2G and BHVO-2G) and a synthetic glass standard (NIST SRM 610). These synthetic glasses were used for instrument calibration and data correction. The sample and standards were placed within 10 mm of the mount center, and the mount's surface was polished to a tolerance of better than 0.5 μm using carborundum sandpaper and emery polishing paste to reduce the effect of sample topography. In order to remove the contamination introduced by sample preparation in the lab, the mounts were washed with ultra-pure water and ethanol alternately for 9 times, using 10 minutes in ultrasonic bath.

Scanning Electron Microscope (SEM) imaging

The glass beads were cleaned with ethanol and carbon-coated before Field Emission Scanning Electron Microprobe (FESEM) analysis. Petrographic characterization of all the glass spherules was made using the ZEISS MERLIN Compact Scanning Electron Microscope at the Beijing SHRIMP Center, Institute of Geology, Chinese Academy of Geological Sciences, Beijing. The SEM used an electron beam current of 6.6 nA and an accelerating voltage of 20 kV to achieve spatially resolved 0.1 μm per pixel back-scatter electron (BSE) and secondary electron (SE) images. After SHRIMP analysis, all U-Pb dating pits were inspected by ZEISS MERLIN FESEM to confirm the position of the analyses and to inspect for the possible unseen inclusions or cracks in the samples.

Electron probe microanalysis (EPMA)

Prior to SHRIMP U-Pb dating chemical compositions of homogeneous areas of the individual glass spherules were obtained using the JEOL JXA-8100 electron microprobe at Institute of Geology, Chinese Academy of Geological Sciences, Beijing, and the JEOL JXA-8230 electron microprobe at Shandong Institute of Geological Sciences, Jinan. A focused electron beam of 20 nA with an acceleration voltage of 15kV and a spot diameter of 15 μm was achieved to examine the major elements of glass spherules and standards. Peak counting times were 20 to 60 s, and backgrounds were counted for half the duration of peak counting time. Accuracy of EPMA analyses was checked by repeated analyses of BCR-2G and BHVO-2G during the whole section. All EPMA data are given in Table S1.

SHRIMP multi-collector U-Pb analysis

The studied polished mounts were cleaned in alcohol and distilled water, and gold coated prior to U-Pb dating. U-Th-Pb data were collected using a SHRIMP IIe MC ion microprobe at the Beijing SHRIMP Center, Institute of Geology, Chinese Academy of Geological Sciences, Beijing. The $^{16}O_2^-$ primary ion beam with a beam intensity of 8-10 nA, a spot size of about 25 μm was produced by filtering via a Wien filter and compressing through a Köhler aperture of 120 μm to analyze glass beads. Due to the low Pb concentrations and unpredictable terrestrial Pb contamination, a

long pre-sputter of 240 seconds was used to raster the surface of an area exceeding analytical spot size by a few micrometres before each analysis. This pre-sputtering removed the gold coating from the area around the analytical spot and limited the effects of surficial contamination. This procedure was followed by automatic centring of the secondary ion beam in the 80-μm source slit for the BCR-2G and BHVO-2G glasses and automatic peak hopping through the magnetic field in sequence from reference mass to Pb isotopes (^{204}Pb, ^{206}Pb, ^{207}Pb, ^{208}Pb), baseline, ^{238}U, ^{232}ThO and $^{238}UO_2$. The secondary ions were measured using a dynamic multi-collector mode with four low-noise channel detection electron multipliers (CDEM). The gain of the four CDEMs was tuned and measured by jumping ^{206}Pb mass of BCR-2G on the different detectors to achieve the gain correction. ^{206}Pb, ^{238}U, ^{232}ThO and $^{238}UO_2$ were measured on the same detector to reduce the effect of the CDEM gain difference. Based on this setting, the dynamic multi-collector technique can achieve higher precision of ^{207}Pb/^{206}Pb and ^{238}U/^{206}Pb than the conventional peak jumping mono-collector approach. Baseline measurements for each Pb peak were performed after analyzing Pb compositions in each spot.

Analyses of BCR-2G (11 μg/g of Pb, 1.69 μg/g of U and 5.90 μg/g of Th) were used to calculate correction factors to account for mass fractionation and relative detector gain calibration. The isotope ratio correction procedure followed Nemchin *el al.* (2020) (*60*) and Che *el al.* (2021) (*22*), and involved dividing each of the recommend isotope ratios for BCR-2G (*61*) by the corresponding average of each ratio obtained from all standards in the session in order to obtain a ratio-specific correction factor that incorporates both mass bias [a few parts per thousand at Pb masses (*62*)] and inter-detector (a few percent) gain. Isotope ratios of unknown samples were then corrected by multiplying by these factors. The U, Th and Pb concentrations were calibrated to BCR-2G. The complete set of BCR-2G analyses corresponding to nine separate analytical sessions is reported in Table S1. Reduction of the SHRIMP data was done manually in Excel. The uncertainties stated for each ratio in the individual sample measurements were derived from the internal run error propagated together with the standard deviations of the BCR-2G analyses for the relevant session and the uncertainty given for the published BCR-2G values (*61*). All analytical data are reported at 1σ in the tables but shown at 2σ in the figures.

To assess the accuracy of the U, Th and Pb isotope analyses and provide additional means to monitor stability of the instrument during the session, BHVO-2G also was run frequently during the analyses of the glass beads. This reference sample has an order-of-magnitude lower Pb concentration compared to BCR-2G (*63*) and approximates the low Pb abundance in many lunar glass beads. The data obtained for BHVO-2G corrected for backgrounds, gains and gains drift are presented in Table S1. These corrected values are within the uncertainties of independently determined ratios (*64*). The data set combines all analyses and the absence of visible statistical change in Pb compositions and U, Th and Pb content between different analytical sessions demonstrates that the gain correction applied based on measurements of the BCR-2G glass is adequate to obtain accurate values for Pb isotopic ratios and U, Th and Pb concentrations in unknown samples.

Approach to U-Pb data reduction and age calculation

General mixing relationships defining isotope compositions of Pb in lunar glasses

The U-Th-Pb ages of lunar materials commonly require resolving complex mixing relationships that define the Pb isotope compositions that reflect in-situ radiogenic decay (*60, 65, 66*). These mixing components can be present in different proportions depending on the nature of the samples and in general are: (i) Pb accumulated *in-situ* as a result of U and Th decay after the formation of

samples, (ii) lunar initial Pb inherited from the sample source and resulting from U decay in this source, (iii) terrestrial Pb resulting from laboratory contamination, when analysing samples returned directly from the Moon (such as Apollo, Luna, and Chang'e samples), and possibly also from alteration on the Earth's surface when investigating lunar meteorites. In addition, some samples such as glasses investigated here can contain (iv) Pb that was mobile in the regolith (*67, 68*) and may have been introduced into the bead after its formation and deposition into the regolith and (v) meteoritic Pb, which is accumulated in lunar soils as a result of continuous addition of asteroid-sourced meteoritic material to the regolith.

Components (ii) -inherited Pb and (iv) -introduced Pb, both representing Pb unsupported by *in-situ* U and Th decay after the formation of samples, can have subtle differences in their isotope compositions, but generally located in the similar parts of Pb isotope diagrams, i.e., top left of $^{207}Pb/^{206}Pb$ vs. $^{204}Pb/^{206}Pb$ plot (Fig. S2). Consequently, correction for these components would result in a similar sharp decrease of $^{207}Pb/^{206}Pb$ and $^{206}Pb/^{238}U$ ages, even when the relative proportion of these components is small. Extra-lunar components (iii) and (v) located at the top-right part of $^{207}Pb/^{206}Pb$ vs. $^{204}Pb/^{206}Pb$ plot (Fig. S2) and correcting measured Pb compositions for either of these components would result in more moderate decrease of $^{207}Pb/^{206}Pb$ and $^{206}Pb/^{238}U$ ages.

Assumptions related to extra-lunar Pb in Chang'e-5 glasses

A number of observations based on the U-Th-Pb data obtained for Chang'e-5 glass population help to make several assumptions related to the mixing relationships existing within glasses from this particular population.

The glasses analyzed show a negative correlation between the ^{206}Pb intensity and $^{204}Pb/^{206}Pb$ ratios (Fig. S3). This correlation suggests an increasing proportion of extra-lunar component(s) in the glasses with low Pb content. This conclusion follows from the known depletion of the Moon in Pb relative to U developed during the first stages of lunar evolution (*65, 69, 70*). As a result of this depletion, lunar materials have low ^{204}Pb abundances and $^{204}Pb/^{206}Pb$ ratios compared to the majority of other Solar System planetary bodies. The trend visible when plotting $^{204}Pb/^{206}Pb$ vs. ^{206}Pb terminates at low 206 cps near the $^{204}Pb/^{206}Pb$ composition of primordial Pb measured in iron meteorites, rather than terrestrial Pb composition (Fig. S3), indicating that this extra-lunar component is represented by asteroid-derived meteoritic material and not a result of laboratory contamination, which cannot explain analyses with highest $^{204}Pb/^{206}Pb$. An extra-lunar meteoritic Pb component is also supported by plotting $^{207}Pb/^{206}Pb$ vs. $^{204}Pb/^{206}Pb$ (Fig. S2), which shows analyses of some glasses located to the right (e.g., higher $^{204}Pb/^{206}Pb$ ratios) of the terrestrial Pb compositions, with about 25% (n=40) of the total analysed populatin having $^{207}Pb/^{206}Pb$ that are too low for a meaningful correction that would result in realistic (positive) age estimates for these glasses. This conclusion is also indirectly supported by the nature of the glass samples and their preparation for analyses. In contrast to many lunar rock samples, which can be contaminated along the grain boundaries and fractures, analyzed areas of the particles analysed here contain homogenous, fracture free, glass. The particles are polished to expose their inner parts and cleaned after polishing as well as pre-spattered prior to analysis to further minimise contamination. Consequently, asteroid derived meteoritic Pb was taken in this study as the best way to account for the extra-lunar Pb present in the analysed glass particles. Assuming further that the population

of meteorites and micro-meteorites responsible for delivering this Pb to the lunar surface is represented by a mixture of different meteorite groups, including iron meteorites, chondrites etc., the Pb composition for correction was estimated to be similar to Pb evolving from primordial Pb in a reservoir with $^{238}U/^{204}Pb$ (μ) of 4, with the uncertainties assigned to include primordial Pb composition. The resulting composition with $^{204}Pb/^{206}Pb$ of 0.0910±0.016 and $^{207}Pb/^{206}Pb$ of 1.03±0.08, used for correction, covers all possible compositions of major meteorite groups.

Possible instrumental issues in the analyses with low Pb count rates

Analyses with low counting rate of Pb [<5 counts per second (cps) of ^{206}Pb] show increasing uncertainties of estimated $^{204}Pb/^{206}Pb$ (Table S1, Fig. S3). Some of these analyses plot to the right of the possible mixed compositions between meteoritic Pb and modern purely radiogenic Pb defined by $^{207}Pb/^{206}Pb$ at zero age equal to 0.046 on $^{207}Pb/^{206}Pb$ vs. $^{204}Pb/^{206}Pb$ diagram (grey area in Fig. S2), although they are within the uncertainties of these possible mixed compositions. Nevertheless, location of these analyses to the right of the mixed compositins indicate possible instrumental excess of ^{204}Pb when the counting rate becomes low. Consistent correction for this ^{204}Pb excess at such low counting rates is not possible. As a result, extreme caution must be taken when using $^{204}Pb/^{206}Pb$ obtained for analyses where count rate is <5 cps of ^{206}Pb. To eliminate potential problems, $^{204}Pb/^{206}Pb$ was not used in any calculations for the glasses with this count rates. All other ratios for these analyses have been used in age calculations and discussion.

Approach to correction for non-in-situ Pb

The set of U-Th-Pb analyses can be grouped into several subsets requiring different methods and assumptions to correction for non-*in-situ* Pb contribution in order to extract reliable ages of the glasses. This also affects the degree of confidence for age estimates made for different groups.

1. The most advanced approach, requiring least number of assumptions can be applied to all analyses of glasses where ^{206}Pb count rates are >5 cps and measured $^{204}Pb/^{206}Pb$ is assumed to be reliable and unaffected by small instrumental excess of ^{204}Pb. Ages of all such glasses can be estimated using a combination of 3D $^{204}Pb/^{206}Pb$-$^{207}Pb/^{206}Pb$-$^{238}U/^{206}Pb$ and $^{206}Pb/^{238}Pb$-$^{207}Pb/^{235}U$-$^{208}Pb/^{232}Th$ systems, irrespective of mixing proportions of different components. The $^{206}Pb/^{238}Pb$-$^{207}Pb/^{235}U$-$^{208}Pb/^{232}Th$ system was used to deal with the common Pb corrections of analyses of terrestrial U-bearing minerals studied by LA-ICP-MS where ^{204}Pb is not analysed (*71*) and where potential resetting of U-Th-Pb system takes place (at time t_2) after the formation of mineral (at time t_1). Nevertheless, it can be adapted to the case of a single formation time with two distinct sources of non-*in-situ* Pb, similar to that of lunar glasses analised here. Assuming that each measured composition of a glass particle represents a mixture of three components: (1) *in-situ* Pb (defining the age of the bead), (2) meteoritic Pb, and (3) unknown lunar Pb, this measured composition will fall on the plane defined by three end-member components. In the $^{204}Pb/^{206}Pb$-$^{207}Pb/^{206}Pb$-$^{238}U/^{206}Pb$ space, it will also fall on the line, lying in this plane and defined by measured composition and meteoritic Pb. This line can be expressed in parametric form as:

$$\frac{^{204}Pb}{^{206}Pb}_{measured} = \frac{^{204}Pb}{^{206}Pb}_{meteroritic} - p \times \frac{^{204}Pb}{^{206}Pb}_{meteroritic} \quad (1)$$

$$\frac{^{207}Pb}{^{206}Pb}_{measured} = \frac{^{207}Pb}{^{206}Pb}_{meteroritic} + p\times\left(\frac{^{207}Pb}{^{206}Pb}_{radiogenic} - \frac{^{207}Pb}{^{206}Pb}_{meteroritic}\right) \quad (2)$$

$$\frac{^{238}U}{^{206}Pb}_{measured} = p\times\frac{^{238}U}{^{206}Pb}_{radiogenic} \quad (3)$$

Here p is an unknown constant, while $(^{207}Pb/^{206}Pb)_{radiogenic}$ and $(^{238}U/^{206}Pb)_{radiogenic}$ represent projection of an analytical point to the concordia plane (^{207}Pb/^{206}Pb-^{238}U/^{206}Pb). This projection will be located on the line representing projection of the original mixing plane to the concordia plane and will intersect the concordia curve at two points corresponding to t_1 and t_2, where t_2 is the age of the glass bead and t_1 does not have a chronological significance but is a reflection of unknown lunar Pb composition that represents non-*in-situ* Pb addition. The line can be expressed as:

$$\frac{\frac{^{238}U}{^{206}Pb}_{radiogenic} - \frac{^{238}U}{^{206}Pb}_{t_1}}{\frac{^{238}U}{^{206}Pb}_{t_2} - \frac{^{238}U}{^{206}Pb}_{t_1}} = \frac{\frac{^{207}Pb}{^{206}Pb}_{radiogenic} - \frac{^{207}Pb}{^{206}Pb}_{t_1}}{\frac{^{207}Pb}{^{206}Pb}_{t_2} - \frac{^{207}Pb}{^{206}Pb}_{t_1}} \quad (4)$$

where

$$\frac{^{238}U}{^{206}Pb}_{t_1} = \frac{1}{\left(e^{\lambda_{238}\times t_1} - 1\right)}$$

$$\frac{^{238}U}{^{206}Pb}_{t_2} = \frac{1}{\left(e^{\lambda_{238}\times t_2} - 1\right)}$$

$$\frac{^{207}Pb}{^{206}Pb}_{t_1} = \frac{\left(e^{\lambda_{235}\times t_1} - 1\right)}{\left(e^{\lambda_{238}\times t_1} - 1\right)}\times\frac{1}{137.88}$$

$$\frac{^{207}Pb}{^{206}Pb}_{t_2} = \frac{\left(e^{\lambda_{235}\times t_2} - 1\right)}{\left(e^{\lambda_{238}\times t_2} - 1\right)}\times\frac{1}{137.88}$$

Combining all equations and rearranging, results in a single equation linking t_1 and t_2:

$$\frac{\frac{^{207}Pb}{^{206}Pb}_{measured}-\frac{^{207}Pb}{^{206}Pb}_{meteroritic}\times\left(1-\frac{\frac{^{204}Pb}{^{206}Pb}_{meteroritic}-\frac{^{204}Pb}{^{206}Pb}_{measured}}{\frac{^{204}Pb}{^{206}Pb}_{meteroritic}}\right)}{\frac{\frac{^{204}Pb}{^{206}Pb}_{meteroritic}-\frac{^{204}Pb}{^{206}Pb}_{measured}}{\frac{^{204}Pb}{^{206}Pb}_{meteroritic}}}$$

$$=\left(\frac{\frac{^{204}Pb}{^{206}Pb}_{meteroritic}-\frac{^{204}Pb}{^{206}Pb}_{measured}}{\frac{^{204}Pb}{^{206}Pb}_{meteroritic}}\times\frac{^{238}U}{^{206}Pb}_{measured}-\frac{1}{\left(e^{\lambda_{238}\times t_1}-1\right)}\right) \tag{5}$$

$$\times\frac{\frac{\left(e^{\lambda_{235}\times t_2}-1\right)}{\left(e^{\lambda_{238}\times t_2}-1\right)}\times\frac{1}{137.88}-\frac{\left(e^{\lambda_{235}\times t_1}-1\right)}{\left(e^{\lambda_{238}\times t_1}-1\right)}\times\frac{1}{137.88}}{\frac{1}{\left(e^{\lambda_{238}\times t_2}-1\right)}-\frac{1}{\left(e^{\lambda_{238}\times t_1}-1\right)}}+\frac{\left(e^{\lambda_{235}\times t_1}-1\right)}{\left(e^{\lambda_{238}\times t_1}-1\right)}\times\frac{1}{137.88}$$

Combining equation (5) with similar equation that can be derived for ^{206}Pb/^{238}Pb-^{207}Pb/^{235}U-^{208}Pb/^{232}Th system and also linking t_1 and t_2 (*71*) results in a system of two equations with two unknown ages t_1 and t_2, which can be resolved numerically. The algorithm for estimation of these two ages for individual glasses was implemented in Matlab and the results are shown in Table S1. A combination of 3D ^{204}Pb/^{206}Pb-^{207}Pb/^{206}Pb-^{238}U/^{206}Pb and ^{206}Pb/^{238}Pb-^{207}Pb/^{235}U-^{208}Pb/^{232}Th systems allows calculation of ages of individual glass beads without prior knowledge of composition of lunar Pb (either inheriter or introduced) unrelated to the *in-situ* U and Th decay after formation of the glasses.

2. Glasses where ^{206}Pb count rates are <5 cps can have instrumental ^{204}Pb excess significant enough to artificially increase ^{204}Pb/^{206}Pb. Consequently, this ratio should not be used for correction of measured Pb composition, which restricts the number of options available for extracting ages of these glasses. Nevertheless, it is probably still valid to use the ^{204}Pb/^{206}Pb-^{207}Pb/^{206}Pb diagram (Fig. S2) to make a rough assessment of mixing relationships between different Pb components in these glasses. On the ^{204}Pb/^{206}Pb-^{207}Pb/^{206}Pb plot (Fig. S2), error crosses representing most analyses of these glasses intersect grey segment, depicting binary mixture between meteoritic Pb and pure radiogenic Pb with ^{207}Pb/^{206}Pb at zero age equal to 0.046. Consequently we made an assumption that these analyses can be explained as binary mixtures without any notable contribution of lunar inherited or introduced Pb.
This assumption allows measured ^{208}Pb/^{206}Pb and ^{232}Th/^{238}U ratios to be used to obtain ages of the glasses. The decay of ^{232}Th and ^{238}U into ^{208}Pb and ^{206}Pb, respectively, can be expressed as:

$$\frac{^{208}Pb}{^{204}Pb}_{measured}=\frac{^{208}Pb}{^{204}Pb}_{meteroritic}+\frac{^{232}Th}{^{204}Pb}_{measured}\times\left(e^{\lambda_{232}\times t}-1\right) \tag{6}$$

$$\frac{^{206}Pb}{^{204}Pb}_{measured} = \frac{^{206}Pb}{^{204}Pb}_{meteroritic} + \frac{^{238}U}{^{204}Pb}_{measured} \times \left(e^{\lambda_{238} \times t} - 1\right) \qquad (7)$$

Which can be re-arranged as:

$$\begin{aligned} \frac{^{208}Pb}{^{206}Pb}_{measured} = \frac{^{208}Pb}{^{206}Pb}_{meteroritic} - \frac{^{208}Pb}{^{206}Pb}_{meteroritic} \times \frac{^{238}U}{^{206}Pb}_{measured} \times \left(e^{\lambda_{238} \times t} - 1\right) \\ + \frac{^{232}Th}{^{238}U}_{measured} \times \frac{^{238}U}{^{206}Pb}_{measured} \times \left(e^{\lambda_{232} \times t} - 1\right) \end{aligned} \qquad (8)$$

and Th/U ages (Table S1) can be determined from (8). Most of these ages (28 analyses out of 31 total with ^{206}Pb <5 cps are younger than 50 Ma. This young age allows testing of binary mixing assumption, as *in-situ* accumulation of ^{207}Pb in such young glasses is extremely small and the measured ^{207}Pb could be viewed as representing pure meteoritic component. With that measured ^{206}Pb/^{238}U could be corrected using ^{207}Pb and ^{206}Pb/^{238}U ages estimated (Table S1). The difference between ^{206}Pb/^{238}U ages and Th/U ages provides some means to determine if assumption of binary mixing holds. Five analyses out of 28 with young Th/U ages also show difference between ^{206}Pb/^{238}U ages and Th/U ages significantly exceeding analytical uncertainties. This difference indicates a significant deviation from assumed two component mixing. The ^{206}Pb/^{238}U ages or Th/U ages of other glasses can be used as the best estimates of ages of these glasses.

This approach can be further tested using 40 analyses of glasses with ^{206}Pb >5 cps and position on ^{204}Pb/^{206}Pb-^{207}Pb/^{206}Pb plot (Fig. S2) indicating that they can also represent two component mixtures. The Th/U ages of these glasses can be determined from equation (8), but ^{206}Pb/^{238}U ages can be obtained by corrcting measured ^{206}Pb/^{238}U using measured ^{204}Pb/^{206}Pb. In addition, ^{204}Pb/^{206}Pb allows correction of measured ^{207}Pb/^{206}Pb. Corrected ^{206}Pb/^{238}U and ^{207}Pb/^{206}Pb, then, allow plotting data on a concordia diagram (Fig. S4) and calculation of concordia ages (*72*). Apparent concordance of correceted data (Fig. S4) and overall similarity of Th/U, ^{206}Pb/^{238}U and concordia ages to 3D ages indicates validity of two component mixing assumption for these 40 glasses and also increases confidence in this approach and the obtained ages.

Consequently, 3D ages of glasses showing ^{206}Pb >5 cps and Th/U ages of glasses with ^{206}Pb <5 cps were used in final compilation of ages of Chang'e-5 glass particles.

Error estimation for U-Th-Pb ages

Calculation of 3D ages and errors was implemented in MatLab (the code is attached at the end of the Supplementary Materials file). Data file "dat_beads.csv" containing ratios obtained for individual glasses was loaded into MatLab. The file contains columns correcponding to ^{204}Pb/^{206}Pb, ^{207}Pb/^{206}Pb, ^{208}Pb/^{206}Pb, ^{238}U/^{206}Pb, ^{232}Th/^{238}U and uncertainties for all ratios estimated by combining analytical uncertainties obtained during SHRIMP analysis of each glass, BCR2G uncertainties determined from multiple analyses of this reference glass during the analytical session and published BCR2G uncertainties for independent determination of these ratios (*61*).

Composition of meteoritic Pb is defined within the MatLab file as a vector accompanied by covariance matrix, defining uncertainties of three Pb ratios and their correlation.

The procedure of estimating ages and errors involves a Monte Carlo approach where 1000 random values for each ratio characterizing glass bead analysis were drawn using each measured ratio and corresponding 1 sigma uncertainty, assuming their normal distribution. A single set of values was also drawn randomly in each simulation to describe meteoritic Pb, using a ratios vector and covariance matrix. As a result 1000 pairs of intercept ages is acquired and the final age and error for each bead is estimated as a mean and 1 standard deviation of these 1000 trials. Two intercept ages and corresponding 1 standard deviations are written in the "final_results" variable.
2D calculations to derive ^{208}Pb corrected Th/U ages as well as ^{208}Pb or ^{208}Pb corrected ^{238}U/^{206}Pb ages and their errors were conducted in a similar manner, but using an excel spreadsheet.

Cratering model approach and assumptions.
Numerical impact simulations were made using the iSALE-2D shock physics code (https://isale-code.github.io/). We simulated five impact crater diameters: 100, 210, 620, 830 and 1300 m, assuming a 7 m thick regolith layer (*25*) over bedrock for the three smaller crater sizes. Melt from the smallest crater modelled here (100 m) is produced almost entirely from the regolith layer whereas the larger craters increasingly sampled the underlying bedrock. Consequently, the two largest craters were modelled with an assumption of a single bedrock layer (Table S3). The 100 m model indicates that craters of this diameter do not penetrate into the bedrock. As a result the effect of two layers is only visible in 200 m model and to a lesser degree in the 600 m calculations. Table S3 shows the impact conditions and target setup. Typical material models were applied for lunar regolith and bedrock, using basalt equation of state for the lunar surface (*73*) and dunite (*74*) for the projectile material. The regolith layer over bedrock was assumed to have 44% porosity [adopted from our recent numerical work (*75*)]. The numerical impact modelling required high numerical resolution to be able to resolve and track the glass spherule-forming material layer. To simulate a fine veneer of ejecta material satisfying spherule-forming conditions in such small craters on the Moon, we used two sets of simulations for the same impact crater. To simulate the ejecta formation, a high-resolution simulation was made (using 40 cells-per-projectile-radius - CPPR) and for simulating up to the transient crater stage, we used a more typical value of 12 CPPR. These runs were used to verify the impact condition and final crater size and morphology. CPPR is a measure of numerical accuracy and for specific simulations, such as early ejection, it is necessary to keep that number high (*75*).
To be considered spherule-forming ejecta, some physical limitations were placed when analysing ejected material. The ejecta temperature of interest was taken to be between 1100 and 2000 K to ensure that melt is created. The initial ejecta velocity vector was calculated from simulations, but then ejecta was assumed to travel ballistically across a flat surface (which is appropriate for the investigated source region of about 150-200 km radius surrounding the landing site). Furthermore, no friction was assumed for ejecta in flight. Altogether, such assumption represents the upper limit for the landing distance calculation. We focus on displaced material and ejecta that matches the temperature and motion/trajectory conditions.
The temperature, speed and ballistic landing distance distributions of the ejecta satisfying the spherule-forming conditions in regional area (up to 200 km distance) can be roughly translated to ejecta mass from the 2D numerical simulation. There is some adjustment necessary to account for numerical simulations being 2D showing a vertical cross-section being projected into 3D space that is symmetric along the vertical axis. To simplify the translation of a 2D simulation into 3D, we only investigated the fraction of the ejecta that is ejected into the spherule-forming layer

compared to the total excavated volume during cratering. For 200 m craters, 8 to 15% of the total transient crater volume satisfied the pre-set spherule forming conditions, and for 500 m, this is the case for 0.5%. However, when transient crater and depth are considered, 500 m craters produce 100 times more in volume/mass compared to 200 m despite the difference in relative melt volumes. Furthermore, the excavation depth for 500 m craters is 5x that of 200 m craters, such that the spherule-forming ejecta from the larger craters comprise 6 times more volume/mass in this case. Therefore, larger craters produce larger amounts of spherule-forming ejecta, and are, therefore, more likely sources. To avoid having to translate 2D simulations into 3D, we instead investigated relative volumes and statistical density of ejecta temperature, speed, and ballistic landing distance. Fig. S5 shows the statistical density of ejecta with distance for all investigated craters and Fig. 4 shows relationships between T of the melted material and transport distance.

Relative probability of presence of glasses produced in different craters at the landing site. Distance of melt transportation can be estimated from the range of initial velocities and ejection angles obtained by modelling and visualized as kernel density estimates (KDE) plots for each of the modelled crater sizes (Fig. S5A). Relative volume of the melt produced in the craters of different size can be also estimated from the model runs and used to adjust/normalize KDEs to these volumes giving relative probability of glasses delivery to a specified distance depending on the model crater size (Fig. S5B). Five separate model runs can be also used as a frame for a more general constraint of probability as a function of crater size and distance (Fig. 5A), which indicates that craters smaller than 500 m and perhaps even those smaller than 1 km in diameter are highly unlikely to produce substantial number of glass beads to register in lunar soil samples in significant proportion. It also suggests that irrespective of the crater size, the majority of glasses would be deposited within 75 km from the crater, with the maximum deposition occurring around 20-50 km. This reconstruction allows an assessment of individual craters identified within the basaltic unit surrounding Chang'e-5 landing site (Table S4) to determine likelihood of glass beads from each of these craters present in Chang'e-5 soil sample. However, there are 29 craters in the database that exceed the largest modelled size of 1300 m, with two of those significantly larger at around 6 and 4 km in diameter. To assess the probability of the glasses formed by these 29 impacts reaching the landing site, the model needs to be extrapolated to at least 6 km crater diameter. This extrapolation is done using relationships between crater size and relative melt volumes established from modelling carters between 100 and 1300 m (Fig. 5C). Using equation for the curve fitting all modelled data (Fig. 5C), relative melt volume produced by a 6 km crater can be estimated and used to extrapolate KDE obtained for a 1300-m crater model to this larger diameter. This extrapolation results in the extension of probability estimates to the larger (up to 6 km) craters (Fig. 5B), which can be used to identify craters near Chang'e-5 landing site with the highest likelihood to produce glasses identified in the soil sample from this site (Table S4). The estimated probability for all craters is shown in relation to crater size in Fig. S6.

Matlab Code for calculation of 3D U-Pb-Th ages and uncertainties

```
%clc, clear all


%input data
data = csvread('data_beads.csv');

for j=1:108 %change to be similar to the number of lines in daya_beads.csv
```

```
for i=1:1000
Pb46m=normrnd(data(j,1),data(j,2));
Pb76m=normrnd(data(j,3),data(j,4));
Pb86m=normrnd(data(j,5),data(j,6));
U38Pb6m=normrnd(data(j,7),data(j,8));
Th32U38=normrnd(data(j,9),data(j,10));

%meteorite or terrestrial composition, copy/paste the relevant
mu=[18.308 15.627 38];%meteorite[11.369 11.582 29.59];terrestrial[18.308 15.627 38]
Sigma=[.78 0.68 1.1; 0.68 0.91 1.1; 1.1 1.1 1.9];%meteorite[1.03 0.63 0.06; 0.63 0.64 0.04; 0.06 0.04
0.061];terrestrial [.78 0.68 1.1; 0.68 0.91 1.1; 1.1 1.1 1.9]
R = mvnrnd(mu,Sigma,1);
Pb64o = R(1,1); %normrnd(11.369,1.03);%9.307;
%Pb64o_err = .1;
Pb74o = R(1,2);%normrnd(11.582,.64);%10.294;
%Pb74o_err = .1;
Pb84o = R(1,3);%normrnd(29.597,.061);%29.476;
%Pb84o_err = .1;

%test(i,1)=Pb64o;
%test(i,2)=Pb74o;
%test(i,3)=Pb84o;

%recalculation of ratios for Th-U-Pb 3D system
Pb86o=Pb84o/Pb64o;
Pb76o=Pb74o/Pb64o;
Pb6U38m=1/U38Pb6m;
Pb7U35m=Pb6U38m*Pb76m*137.88;
Pb8Th32m=Pb86m/U38Pb6m/Th32U38;

% parameters for U-Pb 3D system
A=(Pb76m-Pb74o/Pb64o*(1-(1/Pb64o-Pb46m)*Pb64o))/((1/Pb64o-Pb46m)*Pb64o);
B=U38Pb6m/((1/Pb64o-Pb46m)*Pb64o);
%t2=20;

%solving system of equations
fun = @(t)beads_roots(t,A,B,Pb86o,Pb76o,Pb6U38m,Pb7U35m,Pb8Th32m,Th32U38);
t_i(1) = 5000;
t_i(2) = 1;
t = fsolve(fun,t_i);
Ages(i,1)=t(1);
Ages(i,2)=t(2);
end
aver_ages=mean(Ages,1);
std_ages=std(Ages,1);

final_results(j,1)=aver_ages(1);
final_results(j,2)=std_ages(1);
final_results(j,3)=aver_ages(2);
final_results(j,4)=std_ages(2);
end
```

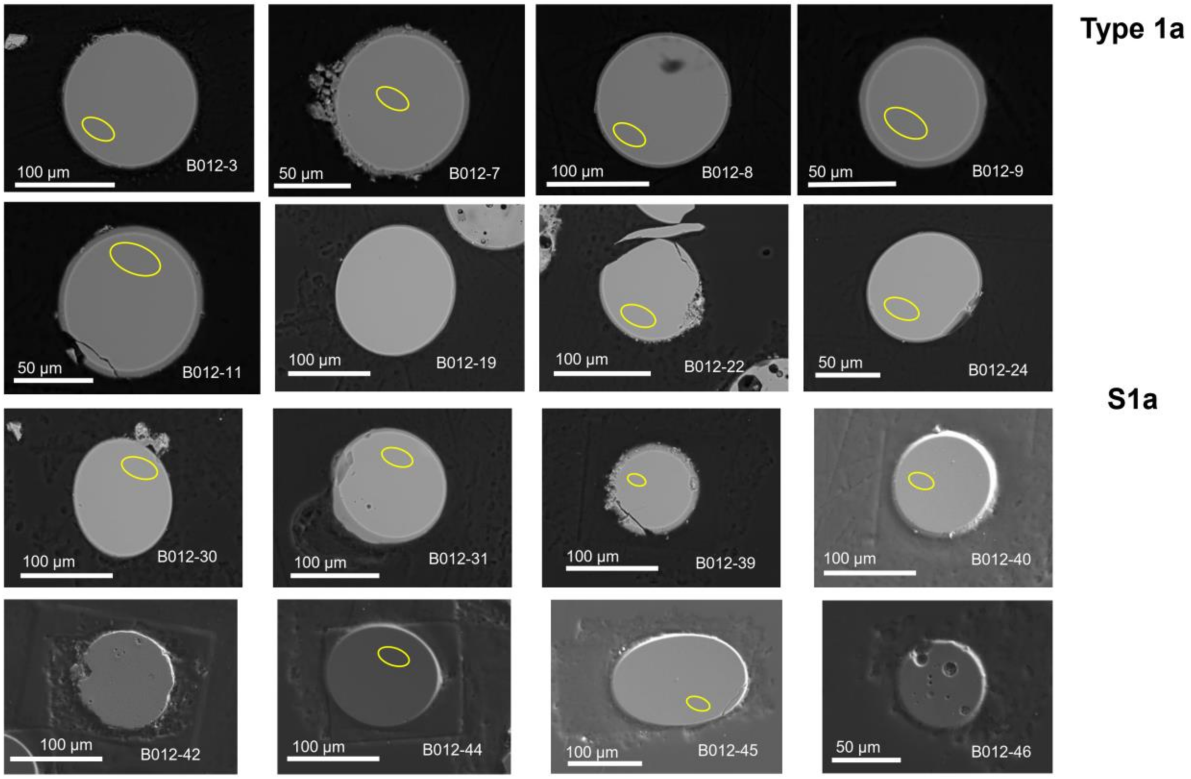
Type 1a
S1a
100 µm
B012-3
50 µm
B012-7
100 µm
B012-8
50 µm
B012-9
50 µm
B012-11
100 µm
B012-19
100 µm
B012-22
50 µm
B012-24
100 µm
B012-30
100 µm
B012-31
100 µm
B012-39
100 µm
B012-40
100 µm
B012-42
100 µm
B012-44
100 µm
B012-45
50 µm
B012-46

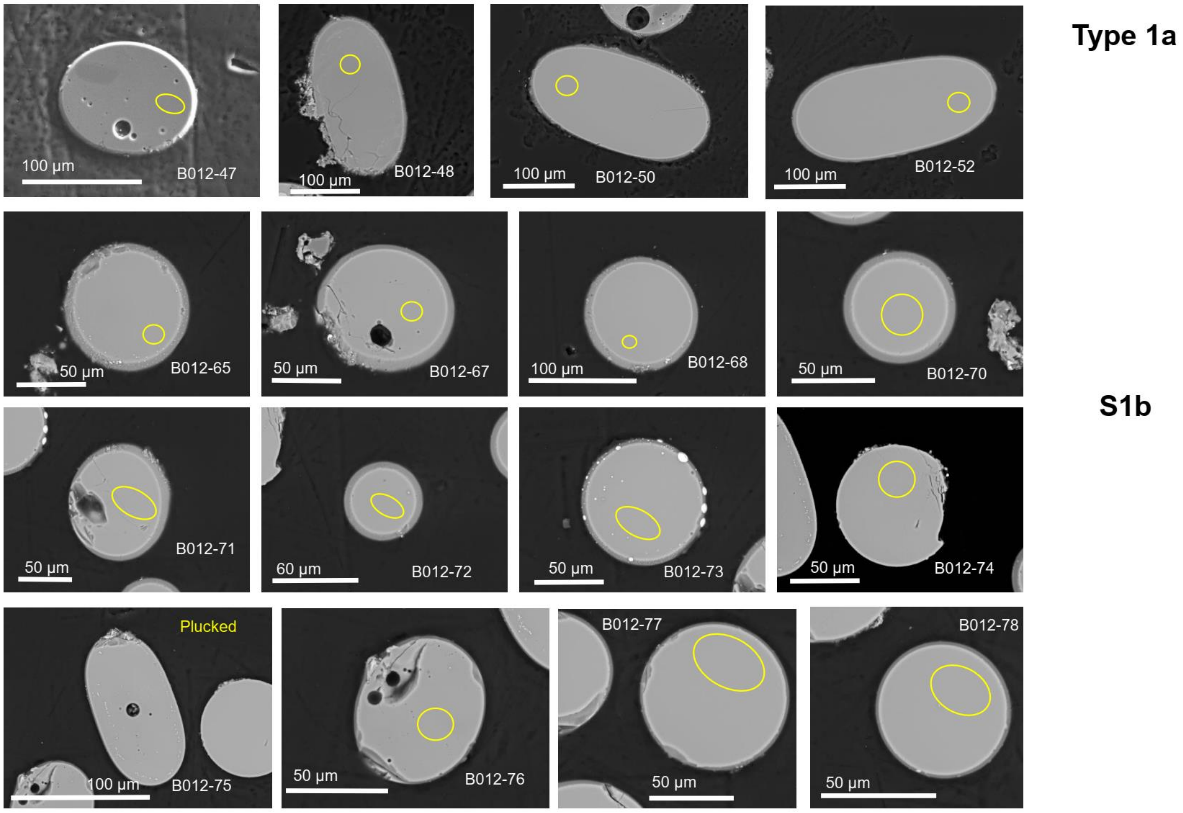

Type 1a
S1b
100 μm
B012-47
100 μm
B012-48
100 μm
B012-50
100 μm
B012-52
50 μm
B012-65
50 μm
B012-67
100 μm
B012-68
50 μm
B012-70
50 μm
B012-71
60 μm
B012-72
50 μm
B012-73
50 μm
B012-74
Plucked
100 μm
B012-75
50 μm
B012-76
B012-77
50 μm
B012-78
50 μm

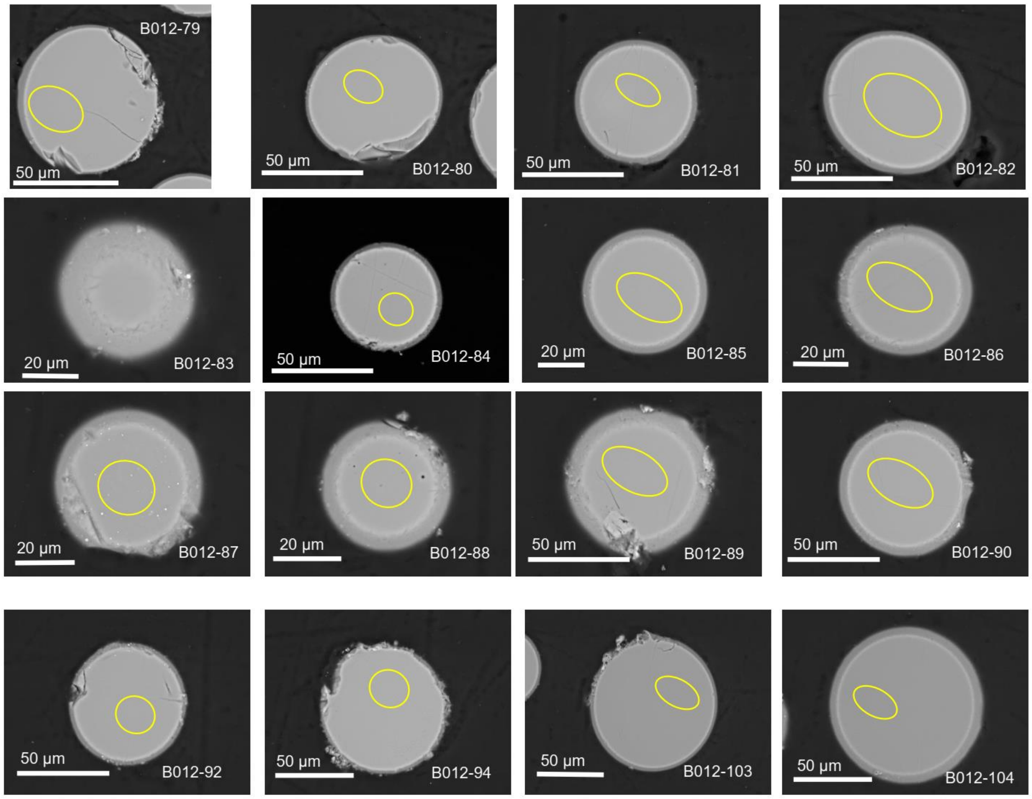
B012-79
50 µm
50 µm
B012-80
50 µm
B012-81
50 µm
B012-82
20 µm
B012-83
50 µm
B012-84
20 µm
B012-85
20 µm
B012-86
20 µm
B012-87
20 µm
B012-88
50 µm
B012-89
50 µm
B012-90
50 µm
B012-92
50 µm
B012-94
50 µm
B012-103
50 µm
B012-104

Type 1a

S1c

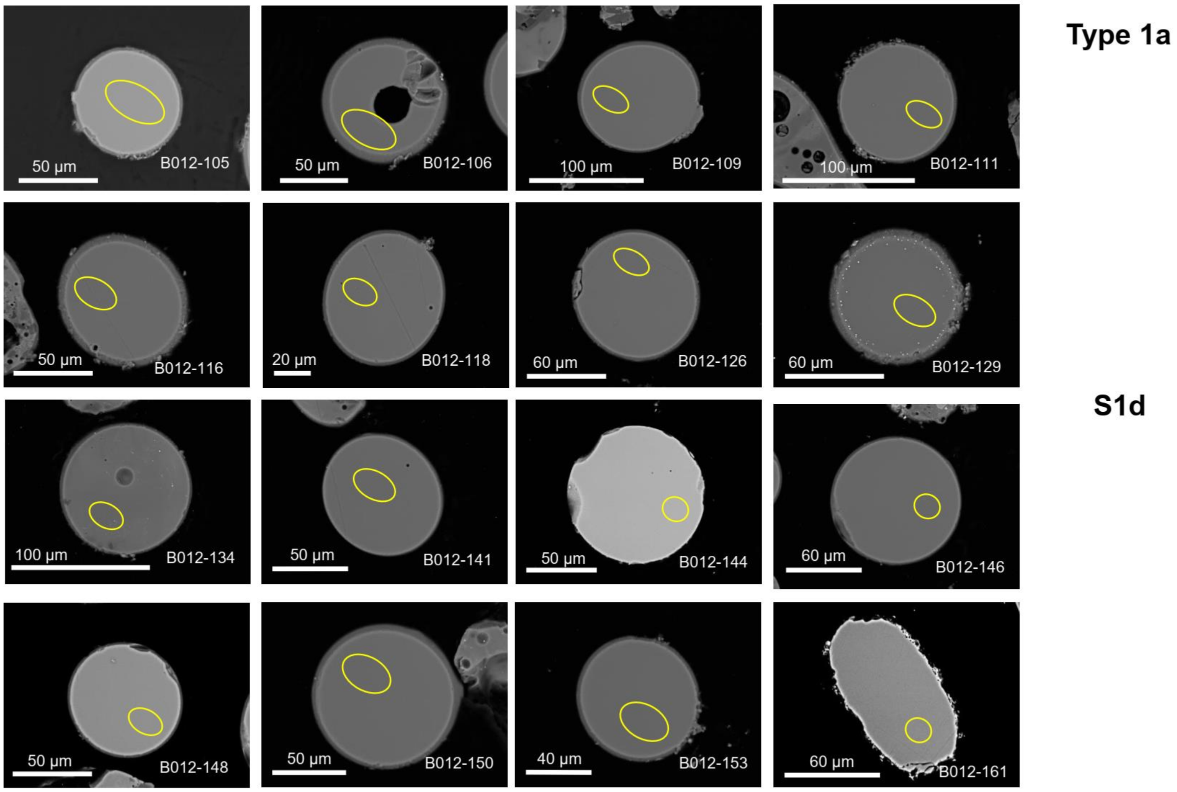

Type 1a
S1d
50 µm
B012-105
50 µm
B012-106
100 µm
B012-109
100 µm
B012-111
50 µm
B012-116
20 µm
B012-118
60 µm
B012-126
60 µm
B012-129
100 µm
B012-134
50 µm
B012-141
50 µm
B012-144
60 µm
B012-146
50 µm
B012-148
50 µm
B012-150
40 µm
B012-153
60 µm
B012-161

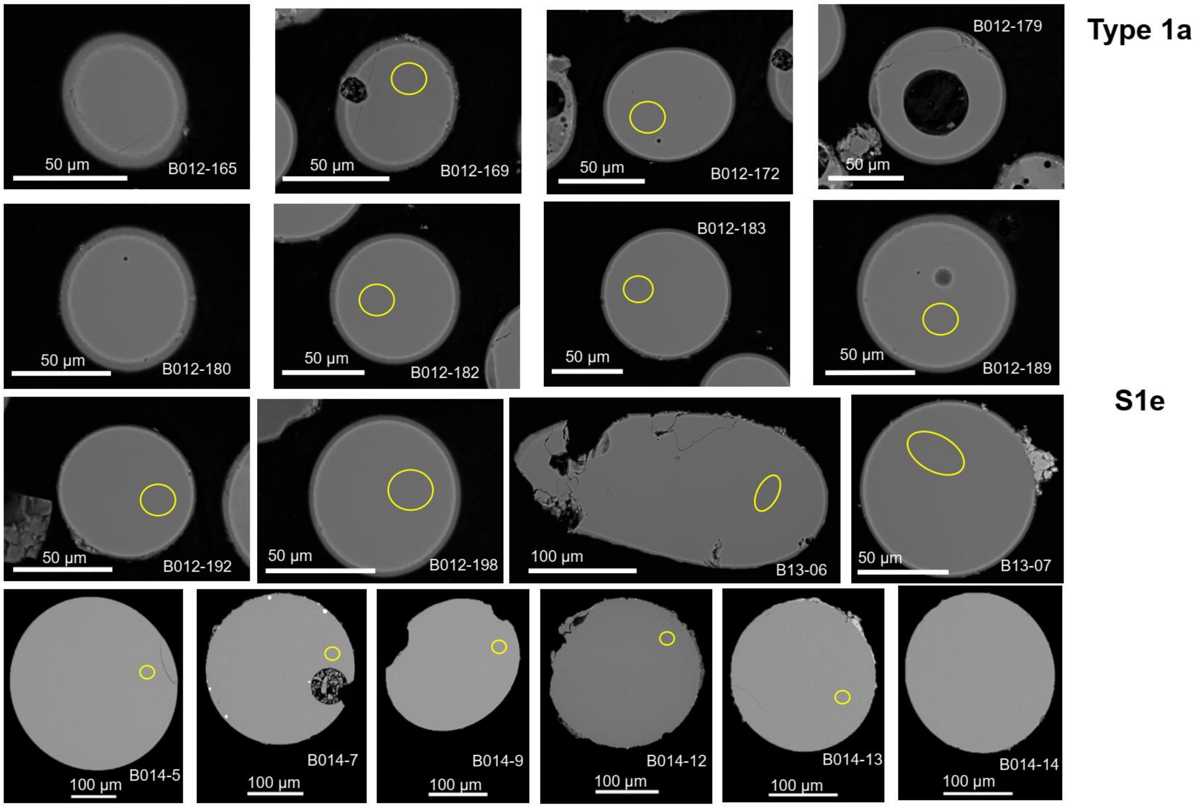
Type 1a
S1e
50 µm
B012-165
50 µm
B012-169
50 µm
B012-172
B012-179
50 µm
50 µm
B012-180
50 µm
B012-182
B012-183
50 µm
50 µm
B012-189
50 µm
B012-192
50 µm
B012-198
100 µm
B13-06
50 µm
B13-07
100 µm
B014-5
B014-7
100 µm
B014-9
100 µm
B014-12
100 µm
B014-13
100 µm
B014-14
100 µm

Type 1a

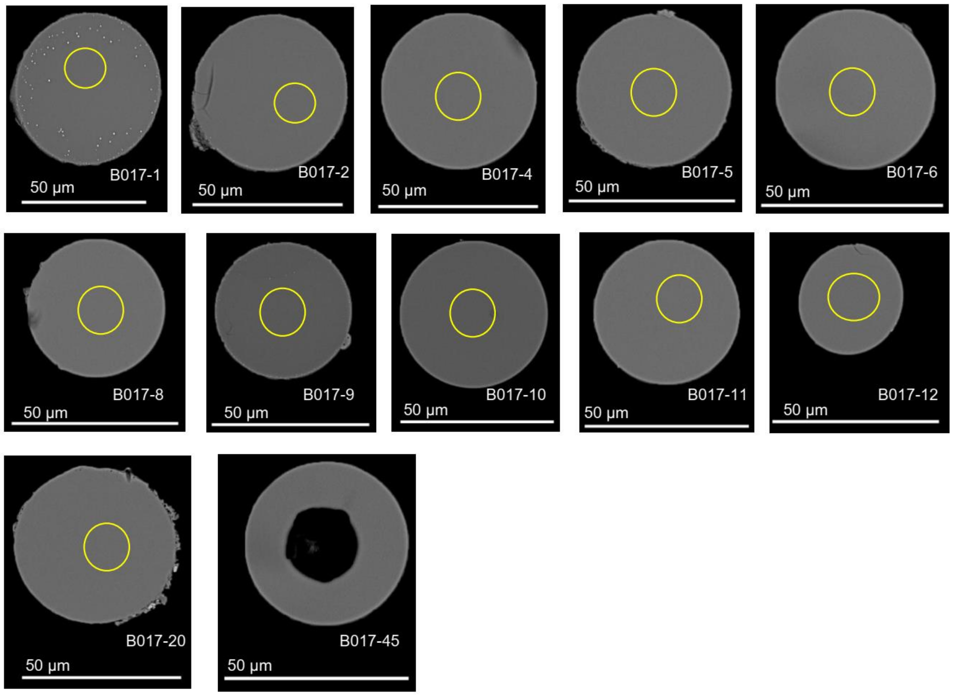
B017-1
50 µm
B017-2
50 µm
B017-4
50 µm
B017-5
50 µm
B017-6
50 µm
B017-8
50 µm
B017-9
50 µm
B017-10
50 µm
B017-11
50 µm
B017-12
50 µm
B017-20
50 µm
B017-45
50 µm

S1f

Type 1b

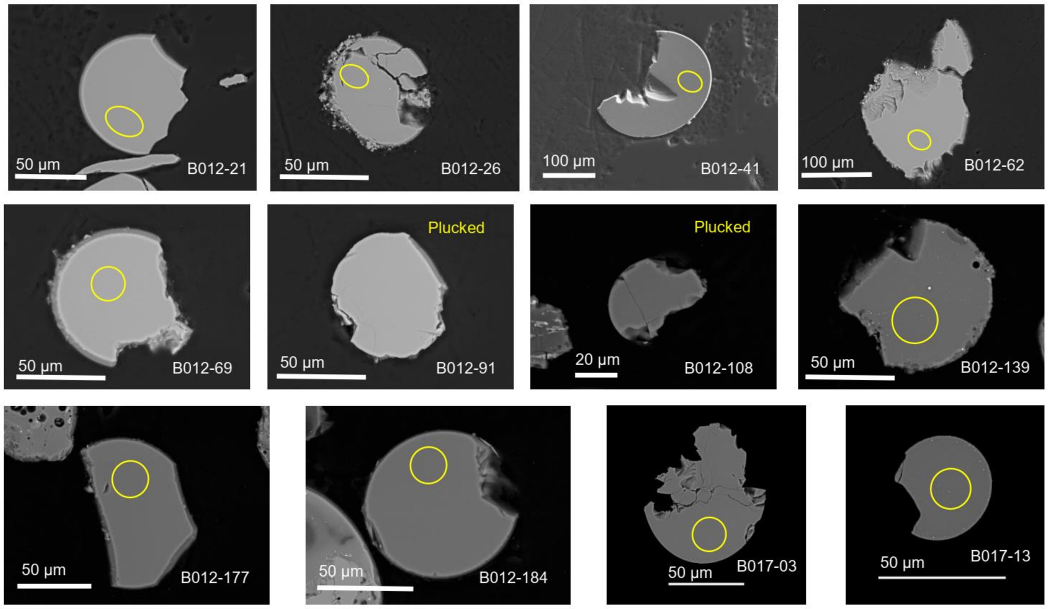
50 µm
B012-21
50 µm
B012-26
100 µm
B012-41
100 µm
B012-62
50 µm
B012-69
Plucked
50 µm
B012-91
Plucked
20 µm
B012-108
50 µm
B012-139
50 µm
B012-177
50 µm
B012-184
50 µm
B017-03
50 µm
B017-13

S1g

Type 1c

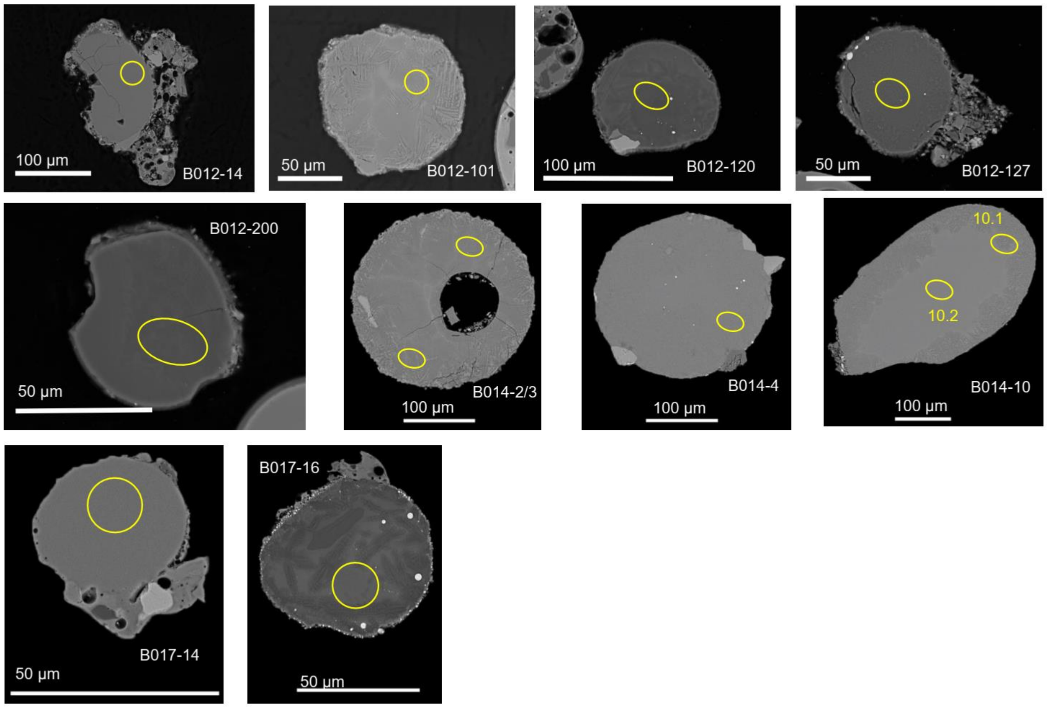
100 µm
B012-14
50 µm
B012-101
100 µm
B012-120
50 µm
B012-127
B012-200
50 µm
100 µm
B014-2/3
B014-4
100 µm
10.1
10.2
100 µm
B014-10
50 µm
B017-14
B017-16
50 µm

S1h

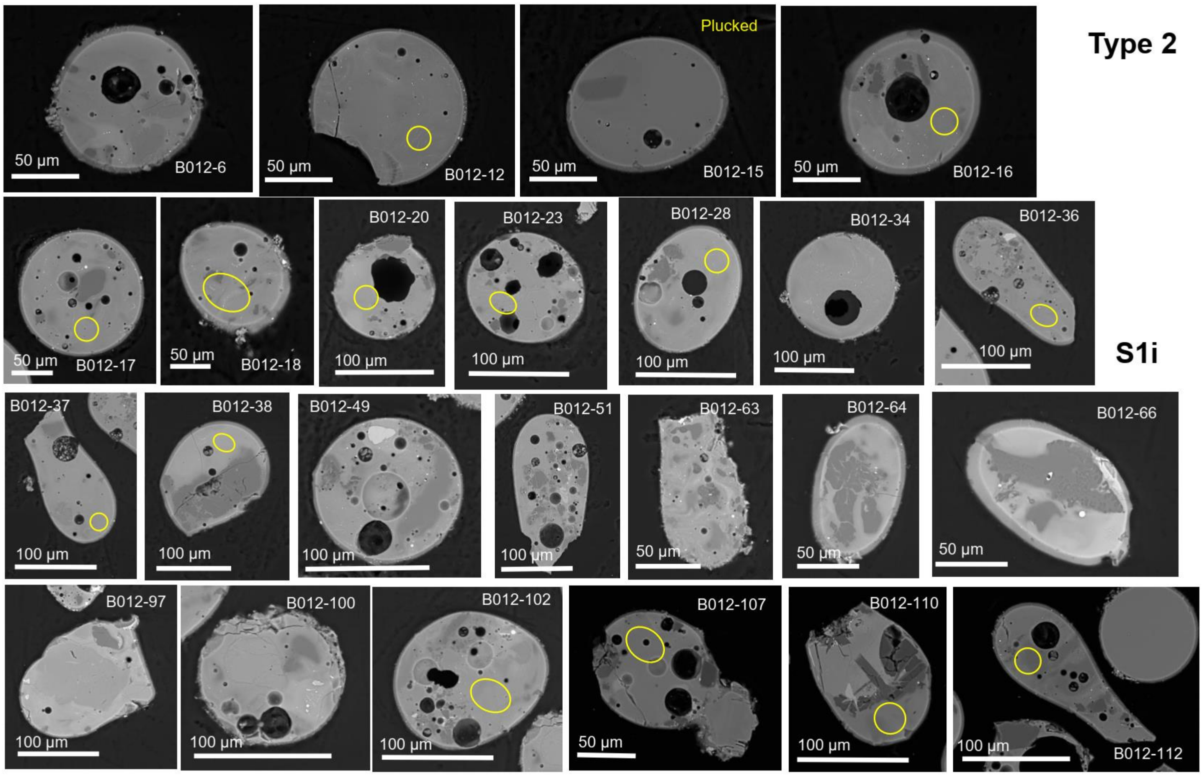
Type 2
S1i
Plucked
50 µm
B012-6
B012-12
B012-15
B012-16
B012-17
B012-18
B012-20
B012-23
B012-28
B012-34
B012-36
100 µm
B012-37
B012-38
B012-49
B012-51
B012-63
B012-64
B012-66
B012-97
B012-100
B012-102
B012-107
B012-110
B012-112

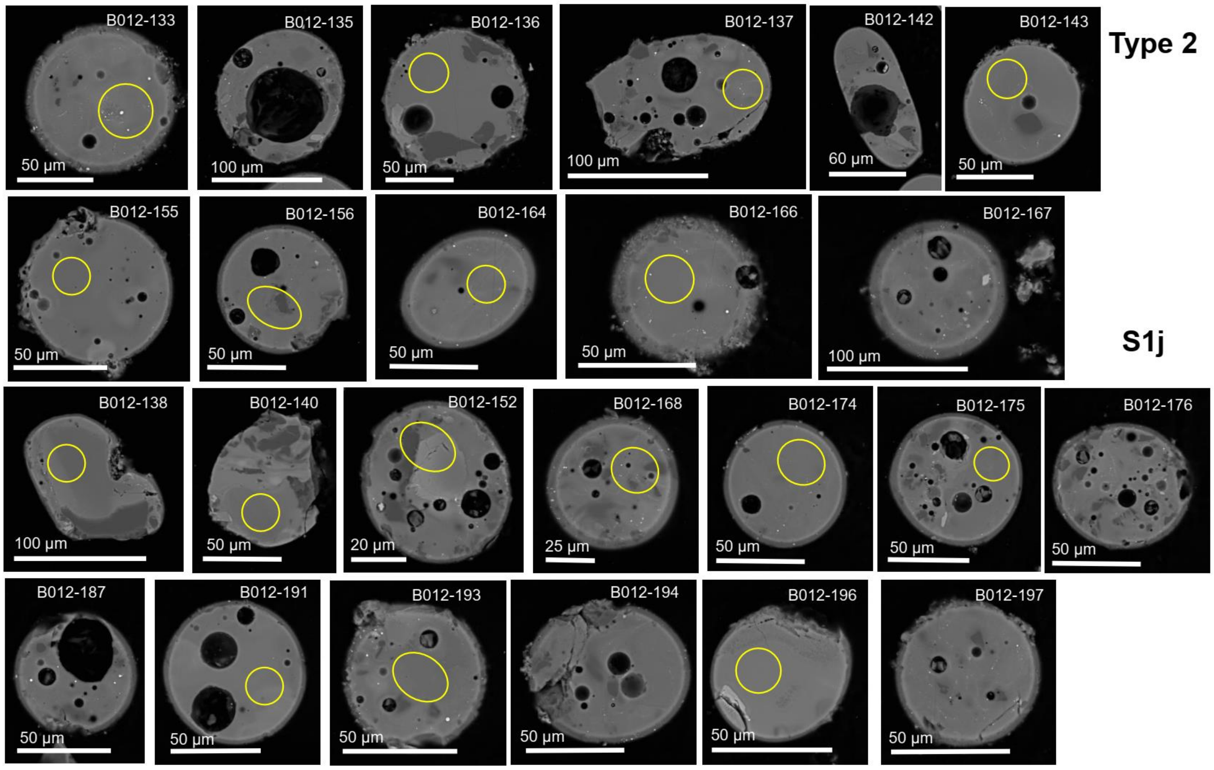
Type 2
B012-133
50 µm
B012-135
100 µm
B012-136
50 µm
B012-137
100 µm
B012-142
60 µm
B012-143
50 µm
B012-155
50 µm
B012-156
50 µm
B012-164
50 µm
B012-166
50 µm
B012-167
100 µm
S1j
B012-138
100 µm
B012-140
50 µm
B012-152
20 µm
B012-168
25 µm
B012-174
50 µm
B012-175
50 µm
B012-176
50 µm
B012-187
50 µm
B012-191
50 µm
B012-193
50 µm
B012-194
50 µm
B012-196
50 µm
B012-197
50 µm

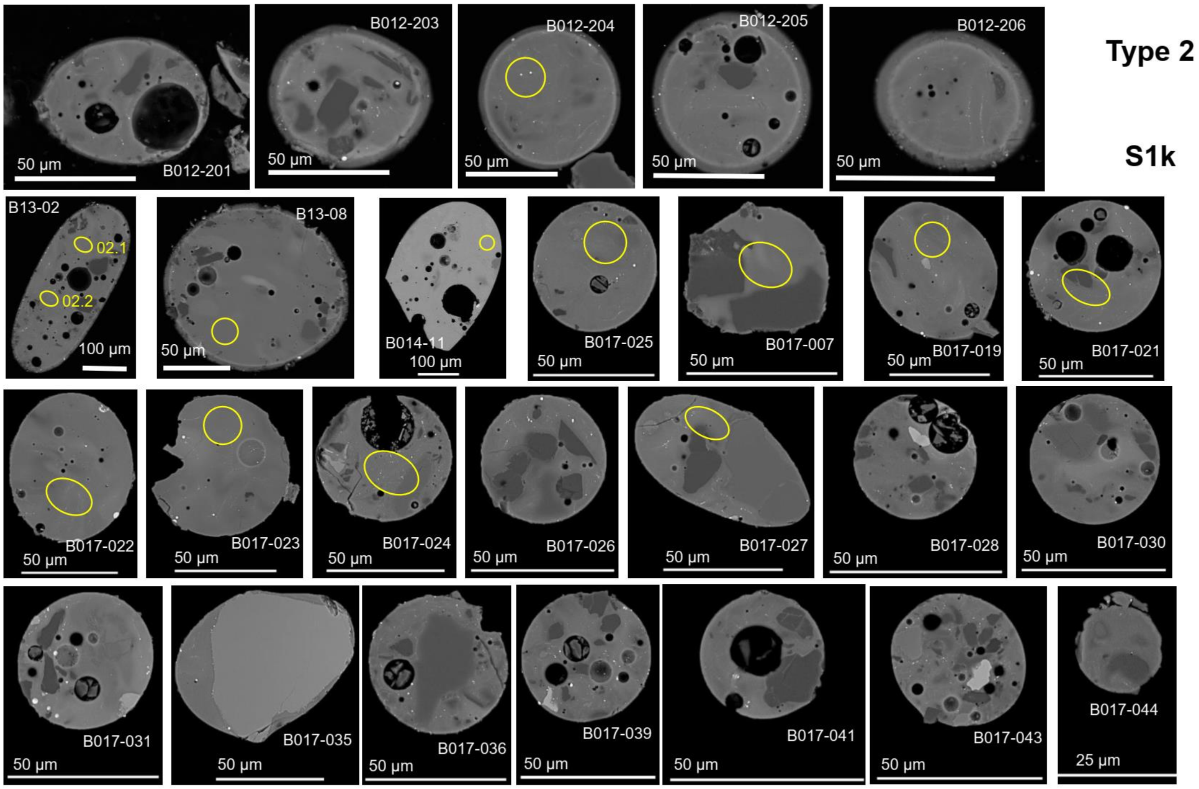
Type 2
S1k
B012-201
50 µm
B012-203
50 µm
B012-204
50 µm
B012-205
50 µm
B012-206
50 µm
B13-02
02.1
02.2
100 µm
B13-08
50 µm
B014-11
100 µm
B017-025
50 µm
B017-007
50 µm
B017-019
50 µm
B017-021
50 µm
B017-022
50 µm
B017-023
50 µm
B017-024
50 µm
B017-026
50 µm
B017-027
50 µm
B017-028
50 µm
B017-030
50 µm
B017-031
50 µm
B017-035
50 µm
B017-036
50 µm
B017-039
50 µm
B017-041
50 µm
B017-043
50 µm
B017-044
25 µm

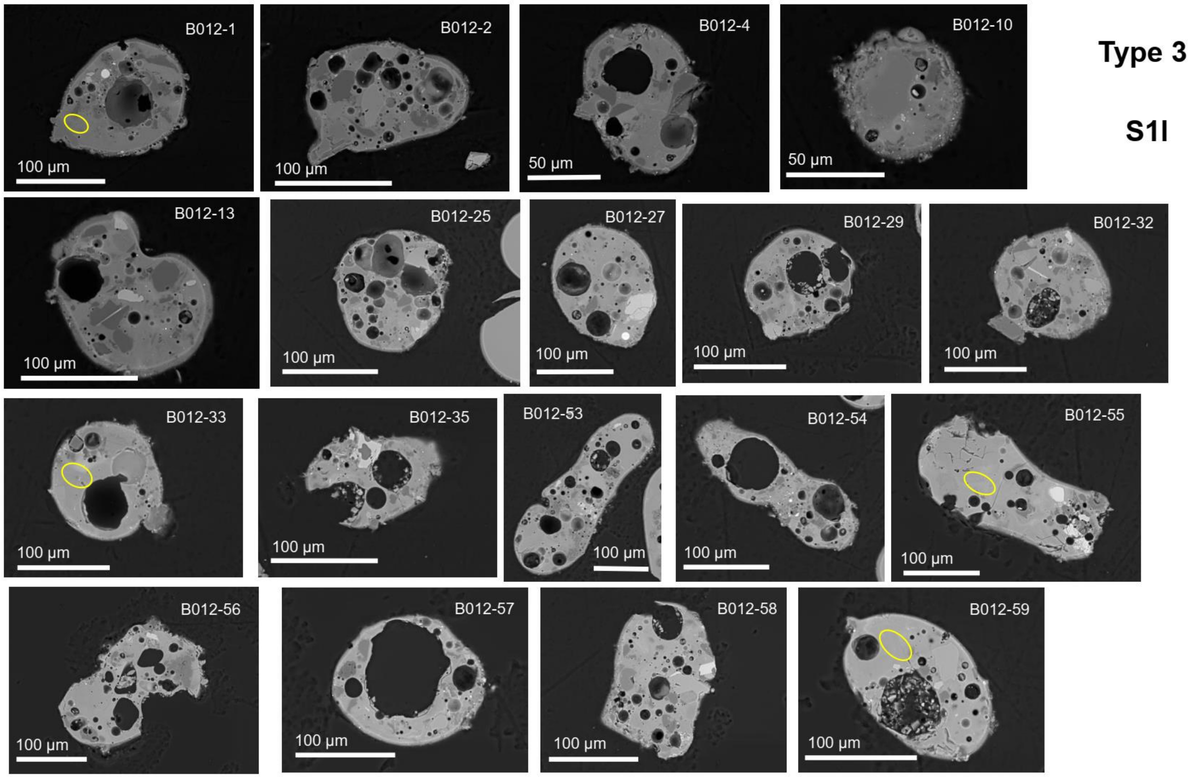
B012-1
100 µm
B012-2
100 µm
B012-4
50 µm
B012-10
50 µm
Type 3
S1l
B012-13
100 µm
B012-25
100 µm
B012-27
100 µm
B012-29
100 µm
B012-32
100 µm
B012-33
100 µm
B012-35
100 µm
B012-53
100 µm
B012-54
100 µm
B012-55
100 µm
B012-56
100 µm
B012-57
100 µm
B012-58
100 µm
B012-59
100 µm

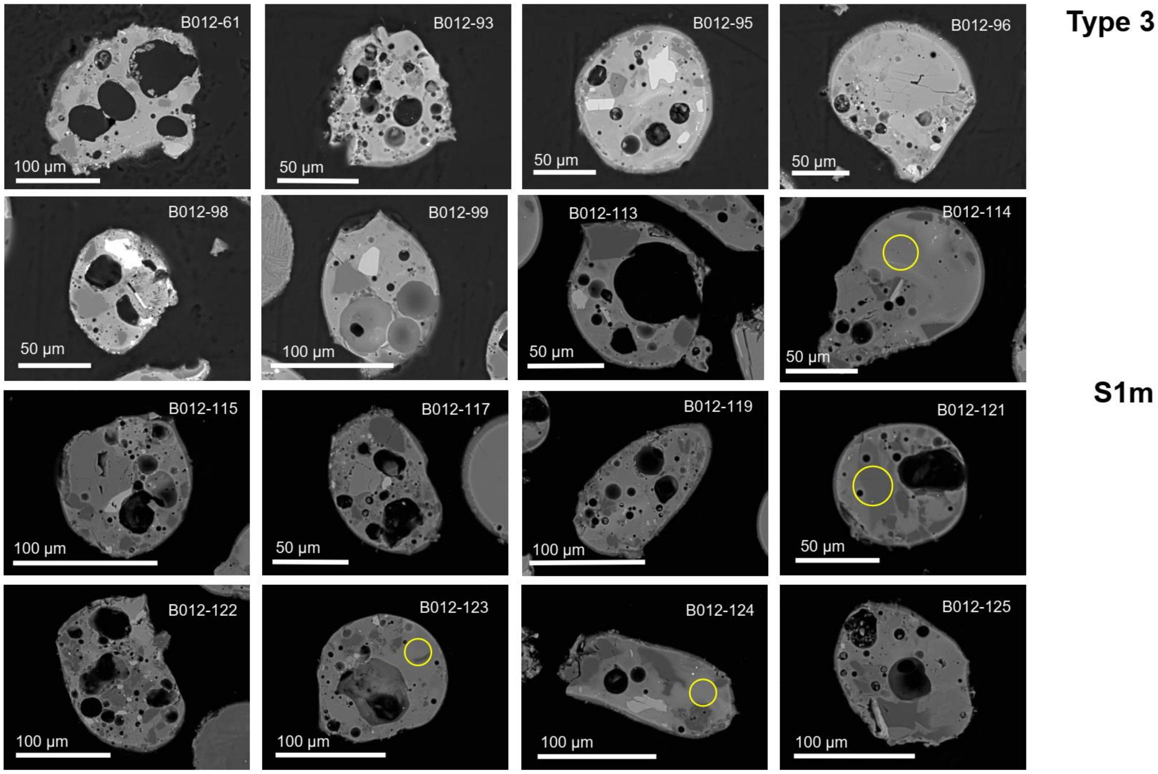
Type 3
S1m
B012-61
100 µm
B012-93
50 µm
B012-95
50 µm
B012-96
50 µm
B012-98
50 µm
B012-99
100 µm
B012-113
50 µm
B012-114
50 µm
B012-115
100 µm
B012-117
50 µm
B012-119
100 µm
B012-121
50 µm
B012-122
100 µm
B012-123
100 µm
B012-124
100 µm
B012-125
100 µm

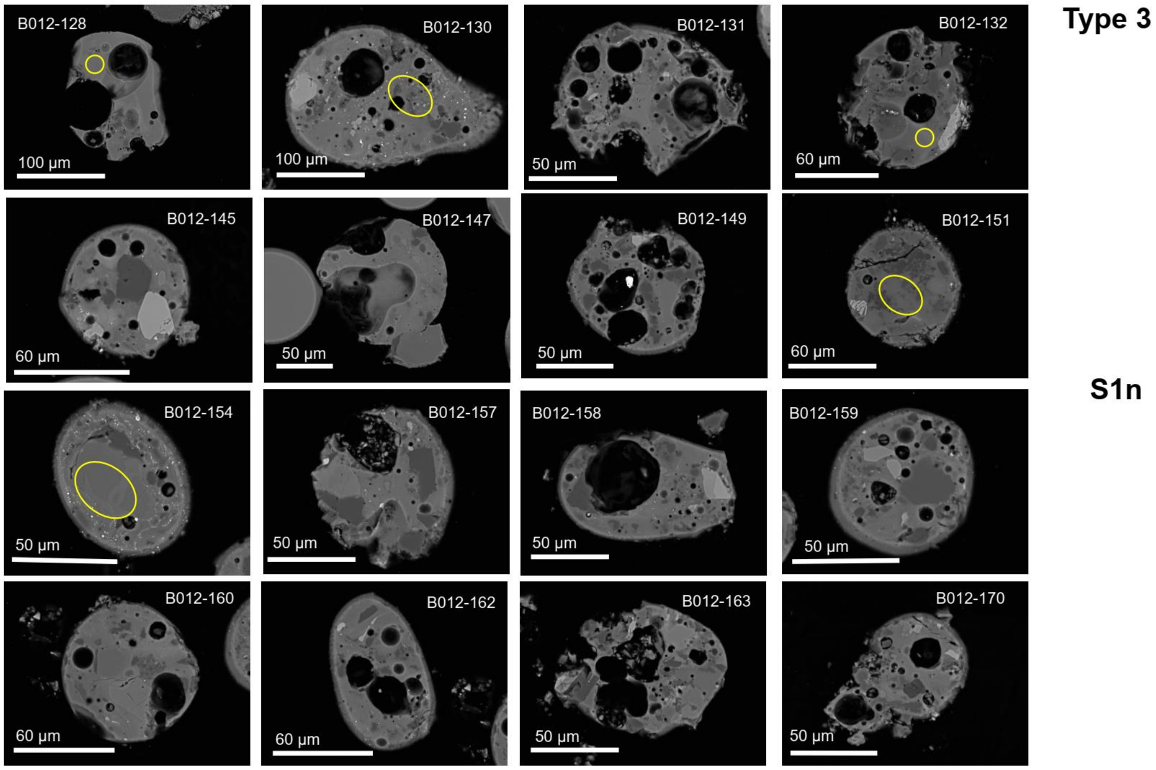
Type 3
S1n
B012-128
100 µm
B012-130
100 µm
B012-131
50 µm
B012-132
60 µm
B012-145
60 µm
B012-147
50 µm
B012-149
50 µm
B012-151
60 µm
B012-154
50 µm
B012-157
50 µm
B012-158
50 µm
B012-159
50 µm
B012-160
60 µm
B012-162
60 µm
B012-163
50 µm
B012-170
50 µm

Type 3

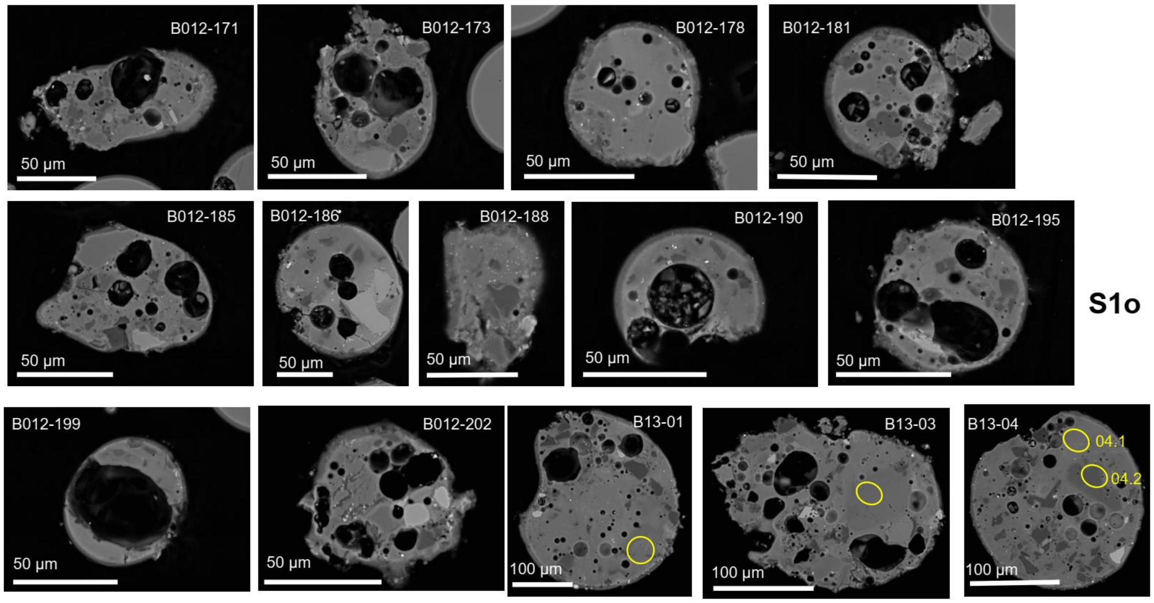

B012-171
50 µm
B012-173
50 µm
B012-178
50 µm
B012-181
50 µm
B012-185
50 µm
B012-186
50 µm
B012-188
50 µm
B012-190
50 µm
B012-195
50 µm
B012-199
50 µm
B012-202
50 µm
B13-01
100 µm
B13-03
100 µm
B13-04
04.1
04.2
100 µm

S1o

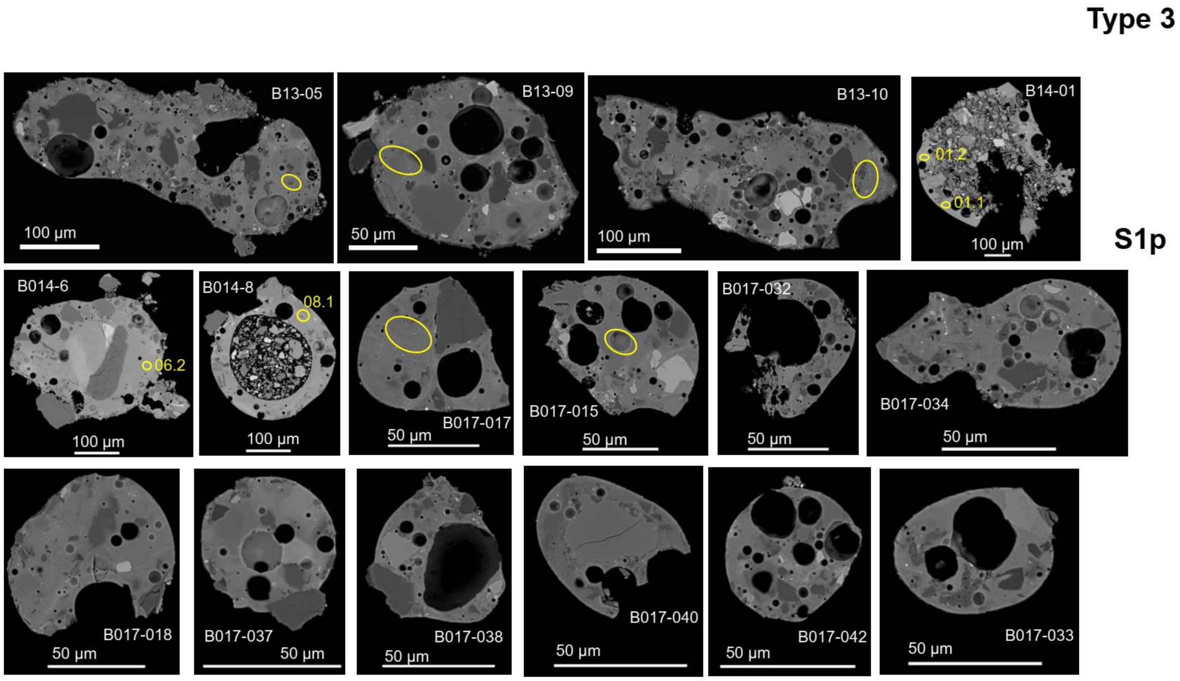

Fig. S1.

Textural classification of Chang'e-5 regolith glasses from sample (CE5C0400YJFM00402) based on backscatter electron imaging (BSE) of individual particles. Categories: Type 1: Homogeneous glasses – entirely molten. Variable abundance and spatial distribution of metal globules. Negligible clasts and few vesicles. A few with large central gas bubbles. 1a -entire melt droplets with continuous quenched exterior surfaces. Shapes include spheres and oblate spheroids. No clasts, occasional vesicles. Variable abundance and distribution of metal blebs. 1b - fragments and broken spheroids with exterior surfaces indicating breakage. 1c - irregularly shaped globules, some with partial crystallization and adhering regolith. Type 2: Largely molten but incompletely homogenised. Aerodynamic shapes indicate individual melt droplets but shapes are more irregular compared to Type 1. Schlieren indicates incomplete melting and imperfectly mixed melt components. Sparse clasts and vesicles occupy modest volume of the particle. Some particles classified as Type 2 have large clasts but relatively dense and homogeneous glass. Type 3: Partially molten. Shapes more irregular than Types 1 and 2 many with poorly defined margins. Abundant clasts, many large vesicles, transitional to regolith agglutinates. Some could be classified as melt-matrix breccias. Types 2 and 3 are transitional but are distinguished visually by their shapes, defined margins, abundances of vesicles and clasts, and angularity of the clasts. Yellow ellipses and circles represent spots analyzed with SHRIMP.

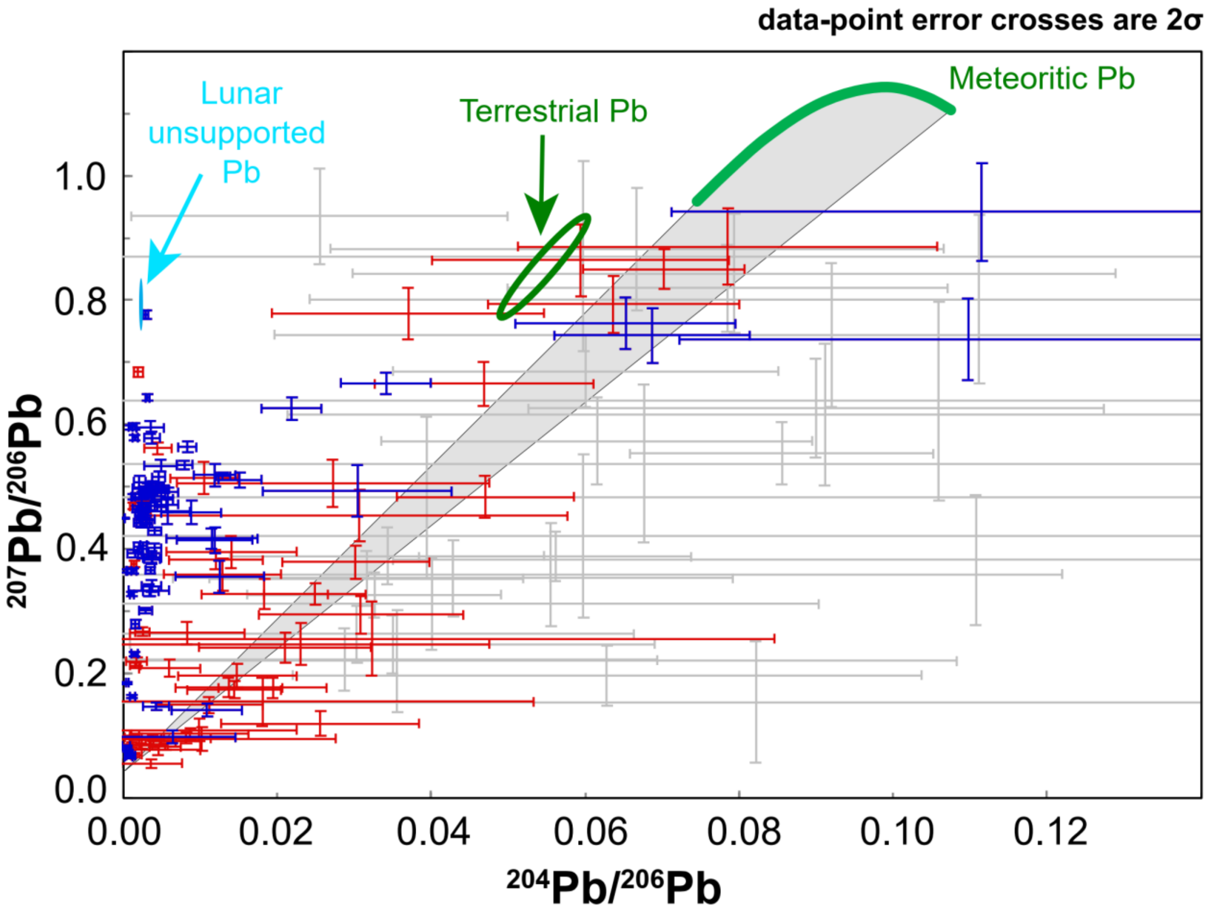

Fig. S2.

$^{204}Pb/^{206}Pb$ vs. $^{207}Pb/^{206}Pb$ plot of analysed glasses. Grey crosses: analyses with ^{206}Pb < 5 cps, Red crosses: analyses of homogenous glasses with ^{206}Pb > 5 cps, Blue crosses: analyses of inhomogeneous glasses with ^{206}Pb < 5 cps. Green curve showing evolution of meteoritic Pb in reservoir with μ- value of 4. Grey area represents mixed compositions between meteoritic Pb and present day purely radiogenic Pb.

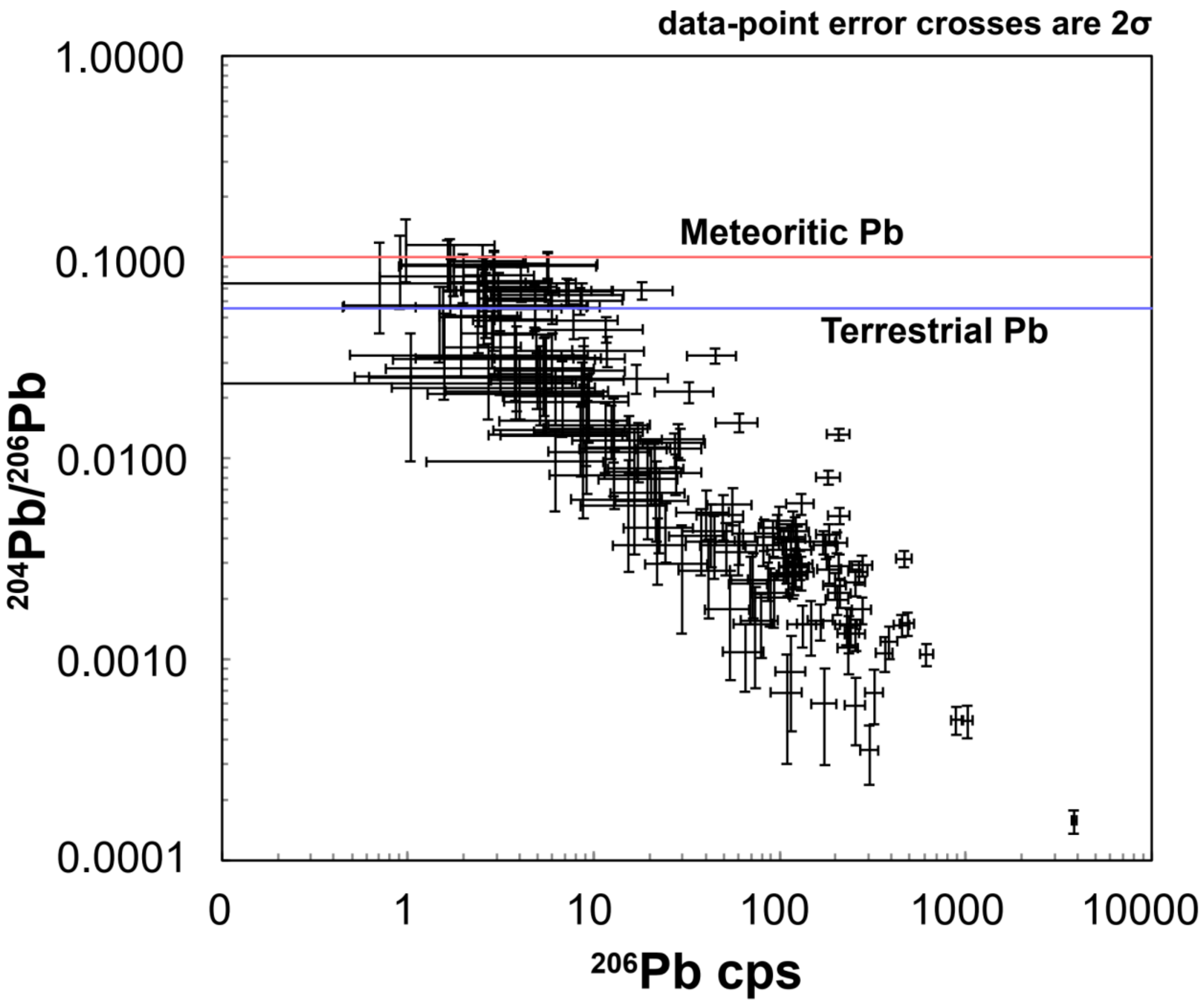

Fig. S3.
^{204}Pb/^{206}Pb vs. ^{206}Pb cps plot of analysed glasses. ^{204}Pb/^{206}Pb of meteoritic Pb and terrestrial Pb are shown as red and blue lines.

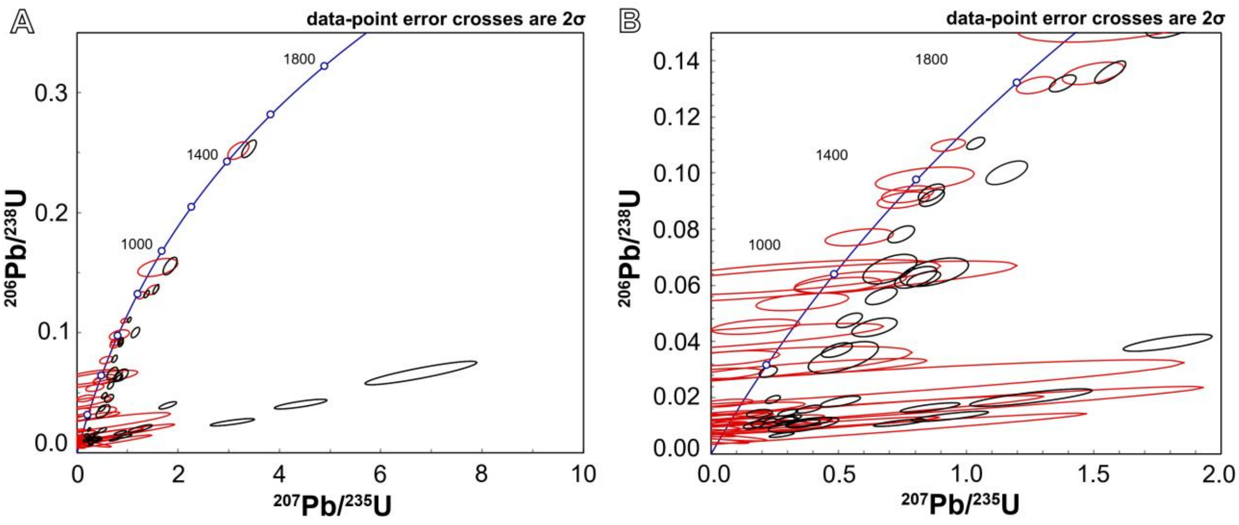

Fig. S4.

Concordia diagram showing results of correction of analyses with $^{206}Pb > 5$ cps that can be viewed as two component mixtures, using meteoritic Pb. Black ellipses: uncorrected data, Red ellipses: corrected data.

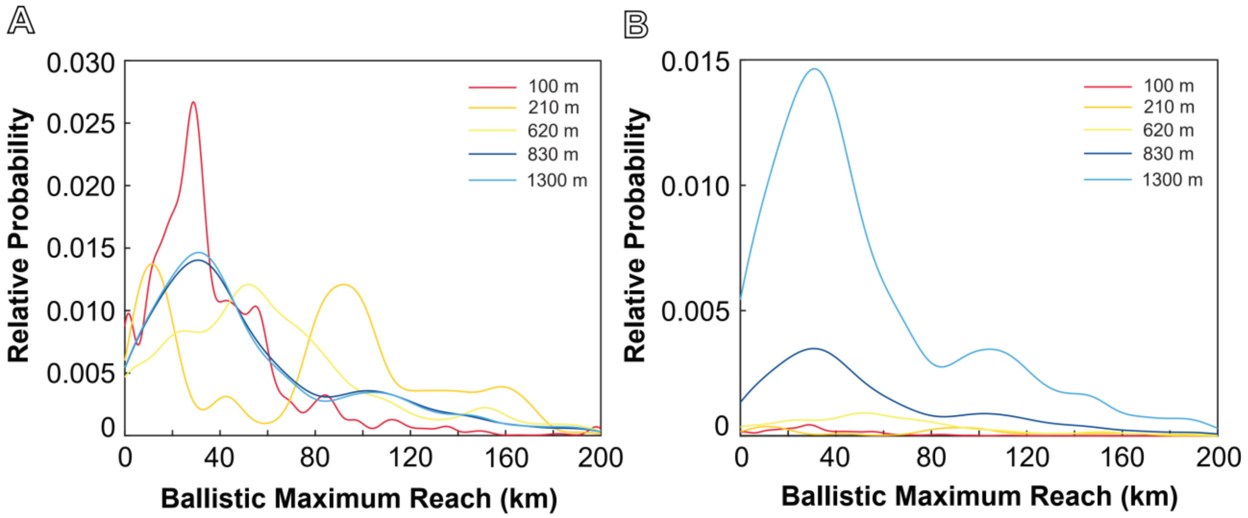

Fig. S5.

Kernel density plots of proportion of the total melt produced in the craters of different sizes as a function of distance from the impact point. Different colours represent separate models for different crater sizes. (A) relative proportions taken directly from each model; (B) relative proportions normalised by the total volume of melt estimated for each model, for relative comparison between the models.

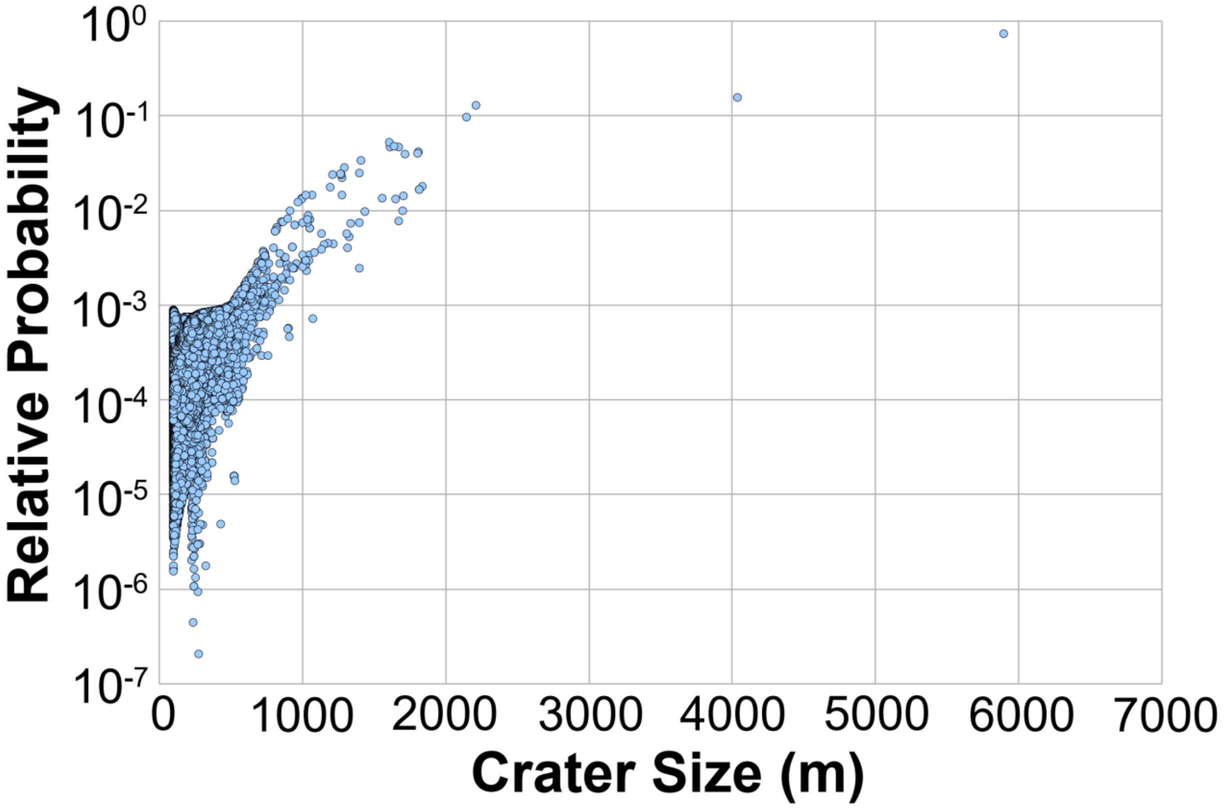

Fig. S6.

Crater size vs. relative probability of presence of glasses from different craters in Chang'e-5 sample calculated for craters larger than 100 m within EM4 unit.

Table S1. (separate file)

Major elements, trace elements, and U-Pb isotope data for glass beads from Chang’e-5 soil.

Table S2. (separate file)

Statistically separate age groups of glasses identified in the population of glass beads from Chang’e-5 soil sample.

Table S3. (separate file)

Numerical impact simulations of impact conditions and cratering results. Here we made 6 detailed high-resolution simulations to demonstrate the ejecta conditions in small lunar craters can meet the spherule-forming physical conditions. We used 2-80-m projectiles in diameter, forming 100-m to 1.3-km diameter craters, assuming the same impact speed and target properties appropriate for the uppermost lunar regolith and bedrock. Excavation depth ranges up to 200 m. The average ballistic reach and ejecta speed is comparable across crater range, given the target and impact speed conditions were the same, which yields ejecta volumes that are heavily dependent on the impactor/crater size. Cell size column indicates the size of each numerical cell (in one dimension), Cell area is the surface area of each numerical cell, Number of cells in spherule layer indicate the number of ejecta cells that meet the spherule-forming conditions (in temperature and ejecta speed), and relative volume in 2D indicates a cross-sectional volumetric measure of the ejecta volume. Since these were 2D simulations, we can only refer to the ejecta volume in relative measures. These are used to determine the statistical likelihood of finding spherules from different size craters surrounding the Chang’e-5 landing site.

Table S4. (separate file)

Chang’e-5 crater database showing size, location, distance to Chang'e-5 landing site and probability of glasses delivery to the site.

REFERENCES AND NOTES

1. J. W. Delano, Pristine lunar glasses: Criteria, data, and implications. *J. Geophys. Res. Solid Earth* **91**, 201–213 (1986).

2. R. A. Zeigler, R. L. Korotev, B. L. Jolliff, L. A. Haskin, C. Floss, The geochemistry and provenance of Apollo 16 mafic glasses. *Geochim. Cosmochim. Acta* **70**, 6050–6067 (2006).

3. M. D. Norman, K. J. D. Adena, A. G. Christy, Provenance and Pb isotopic ages of lunar volcanic and impact glasses from the Apollo 17 landing site. *Aust. J. Earth Sci.* **59**, 291–306 (2012).

4. N. E. B. Zellner, Lunar Impact Glasses: Probing the Moon's surface and constraining its impact history. *J. Geophys. Res. Planets* **124**, 2686–2702 (2019).

5. T. S. Culler, T. A. Becker, R. A. Muller, P. R. Renne, Lunar Impact History from $^{40}Ar/^{39}Ar$ dating of glass spherules. *Science* **287**, 1785–1788 (2000).

6. J. Levine, T. A. Becker, R. A. Muller, P. R. Renne, $^{40}Ar/^{39}Ar$ dating of Apollo 12 impact spherules. *Geophys. Res. Lett.* **32**, L15201 (2005).

7. N. E. B. Zellner, J. W. Delano, T. D. Swindle, F. Barra, E. Olsen, D. C. B. Whittet, Evidence from $^{40}Ar/^{39}Ar$ ages of lunar impact glasses for an increase in the impact rate ∼800Ma ago. *Geochim. Cosmochim. Acta* **73**, 4590–4597 (2009).

8. N. E. B. Zellner, J. W. Delano, T. D. Swindle, F. Barra, E. Olsen, D. C. B. Whittet, Apollo 17 regolith, 71501,262: A record of impact events and mare volcanism in lunar glasses. *Meteoritics Planet. Sci.* **44**, 839–851 (2009).

9. V. A. Fernandes, J. Fritz, B. P. Weiss, I. Garrick-Bethell, D. L. Shuster, The bombardment history of the Moon as recorded by ^{40}Ar-^{39}Ar chronology. *Meteoritics Planet. Sci.* **48**, 241–269 (2013).

10. N. E. B. Zellner, J. W. Delano, $^{40}Ar/^{39}Ar$ ages of lunar impact glasses: Relationships among Ar diffusivity, chemical composition, shape, and size. *Geochim. Cosmochim. Acta* **161**, 203–218 (2015).

11. N. E. B. Zellner, Cataclysm no more: New views on the timing and delivery of lunar impactors. *Origins Life Evol. Biospheres* **47**, 261–280 (2017).

12. A. A. Nemchin, M. J. Whitehouse, M. L. Grange, J. R. Muhling, On the elusive isotopic composition of lunar Pb. *Geochim. Cosmochim. Acta* **75**, 2940–2964 (2011).

13. A. A. Nemchin, M. D. Norman, M. L. Grange, R. A. Zeigler, M. J. Whitehouse, J. R. Muhling, R. Merle, U-Pb isotope systematics and impact ages recorded by a chemically diverse population of glasses from an Apollo 14 lunar soil. *Geochim. Cosmochim. Acta* **321**, 206–243 (2022).

14. C. K. Shearer, J. J. Papike, Basaltic magmatism on the Moon: A perspective from volcanic picritic glass beads. *Geochim. Cosmochim. Acta* **57**, 4785–4812 (1993).

15. A. E. Saal, E. H. Hauri, M. L. Cascio, J. A. Van Orman, M. C. Rutherford, R. F. Cooper, Volatile content of lunar volcanic glasses and the presence of water in the Moon's interior. *Nature* **454**, 192–195 (2008).

16. N. E. B. Zellner, P. D. Spudis, J. W. Delano, D. C. B. Whittet, Impact glasses from the Apollo 14 landing site and implications for regional geology. *J. Geophys. Res. Planets* **107**, 5102 (2002).

17. J. W. Delano, N. E. B. Zellner, F. Barra, E. Olson, T. D. Swindle, N. J. Tibbetts, D. C. B. Whittet, An integrated approach to understanding Apollo 16 impact glasses: Chemistry, isotopes, and shape. *Meteoritics Planet. Sci.* **42**, 993–1004 (2007).

18. R. L. Korotev, R. A. Zeigler, C. Floss, On the origin of impact glass in the Apollo 16 regolith. *Geochim. Cosmochim. Acta* **74**, 7362–7388 (2010).

19. W. K. Hartmann, C. Quantin, N. Mangold, Possible long-term decline in impact rates: 2. Lunar impact-melt data regarding impact history. *Icarus* **186**, 11–23 (2007).

20. Y.-H. Huang, D. A. Minton, N. E. B. Zellner, M. Hirabayashi, J. E. Richardson, C. I. Fassett, No change in the recent lunar impact flux required based on modeling of impact glass spherule age distributions. *Geophys. Res. Lett.* **45**, 6805–6813 (2018).

21. C. Zhou, Y. Jia, J. Liu, H. Li, Y. Fan, Z. Zhang, Y. Liu, Y. Jiang, B. Zhou, Z. He, J. Yang, Y. Hu, Z. Liu, L. Qin, B. Lv, Z. Fu, J. Yan, C. Wang, Y. Zou, Scientific objectives and payloads of the lunar sample return mission—Chang'E-5. *Adv. Space Res.* **69**, 823–836 (2022).

22. X. Che, A. Nemchin, D. Liu, T. Long, C. Wang, M. D. Norman, K. H. Joy, R. Tartese, J. Head, B. Jolliff, J. F. Snape, C. R. Neal, M. J. Whitehouse, C. Crow, G. Benedix, F. Jourdan, Z. Yang, C. Yang, J. Liu, S. Xie, Z. Bao, R. Fan, D. Li, Z. Li, S. G. Webb, Age and composition of young basalts on the Moon, measured from samples returned by Chang'e-5. *Science* **374**, 887–890 (2021).

23. Q.-L. Li, Q. Zhou, Y. Liu, Z. Xiao, Y. Lin, J.-H. Li, H.-X. Ma, G.-Q. Tang, S. Guo, X. Tang, J.-Y. Yuan, J. Li, F.-Y. Wu, Z. Ouyang, C. Li, X.-H. Li, Two-billion-year-old volcanism on the Moon from Chang'e-5 basalts. *Nature* **600**, 54–58 (2021).

24. Y. Qian, L. Xiao, J. W. Head, C. H. van der Bogert, H. Hiesinger, L. Wilson, Young lunar mare basalts in the Chang'e-5 sample return region, northern Oceanus Procellarum. *Earth Planet. Sci. Lett.* **555**, 116702 (2021).

25. Y. Qian, L. Xiao, Q. Wang, J. W. Head, R. Yang, Y. Kang, C. H. van der Bogert, H. Hiesinger, X. Lai, G. Wang, Y. Pang, N. Zhang, Y. Yuan, Q. He, J. Huang, J. Zhao, J. Wang, S. Zhao, China's Chang'e-5 landing site: Geology, stratigraphy, and provenance of materials. *Earth Planet. Sci. Lett.* **561**, 116855 (2021).

26. J. W. Head, L. Wilson, Rethinking lunar mare basalt regolith formation: New concepts of lava flow protolith and evolution of regolith thickness and internal structure. *Geophys. Res. Lett.* **47**, e2020GL088334 (2020).

27. F. Hörz, R A Muller, T A Becker, T S Culler, D B Karner, P R Renne, Time-variable cratering rates? *Science* **288**, 2095–2095 (2000).

28. C. Li, H. Hu, M.-F. Yang, Z.-Y. Pei, Q. Zhou, X. Ren, B. Liu, D. Liu, X. Zeng, G. Zhang, H. Zhang, J. Liu, Q. Wang, X. Deng, C. Xiao, Y. Yao, D. Xue, W. Zuo, Y. Su, W. Wen, Z. Ouyang, Characteristics of the lunar samples returned by Chang'E-5 mission. *Natl. Sci. Rev.* **9**, nwab188 (2022).

29. M. T. Naney, D. M. Crowl, J. J. Papike, The Apollo 16 drill core: Statistical analysis of glass chemistry and the characterization of a high alumina-silica poor (HASP) glass. *Lunar Planet. Sci. Conf. Proc.* **1**, 155–184 (1976).

30. C. J. Renggli, P. L. King, R. W. Henley, M. D. Norman, Volcanic gas composition, metal dispersion and deposition during explosive volcanic eruptions on the Moon. *Geochim. Cosmochim. Acta* **206**, 296–311 (2017).

31. G. Gehrels, Detrital Zircon U-Pb Geochronology: Current Methods and New Opportunities, in *Tectonics of Sedimentary Basins* (Blackwell Publishing Ltd, 2012), pp. 45–62.

32. M. S. Sambridge, W. Compston, Mixture modeling of multi-component data sets with application to ion-probe zircon ages. *Earth Planet. Sci. Lett.* **128**, 373–390 (1994).

33. H. J. Melosh, A. M. Vickery, Melt droplet formation in energetic impact events. *Nature* **350**, 494–497 (1991).

34. B. C. Johnson, "The formation of distal impact ejecta," thesis, Purdue University, West Lafayette (2013).

35. P. H. Warren, Lunar rock-rain: Diverse silicate impact-vapor condensates in an Apollo-14 regolith breccia. *Geochim. Cosmochim. Acta* **72**, 3562–3585 (2008).

36. O. I. Yakovlev, M. V. Gerasimov, Y. P. Dikov, Estimation of temperature conditions for the formation of HASP and GASP glasses from the lunar regolith. *Geochem. Int.* **49**, 213 (2011), 223.

37. Y. Qian, L. Xiao, S. Yin, M. Zhang, S. Zhao, Y. Pang, J. Wang, G. Wang, J. W. Head, The regolith properties of the Chang'e-5 landing region and the ground drilling experiments using lunar regolith simulants. *Icarus* **337**, 113508 (2020).

38. N. Artemieva, B. Ivanov, Launch of martian meteorites in oblique impacts. *Icarus* **171**, 84–101 (2004).

39. J. R. Elliott, H. J. Melosh, B. C. Johnson, The role of target strength on the ejection of martian meteorites. *Icarus* **375**, 114869 (2022).

40. M. D. Norman, F. Jourdan, S. S. M. Hui, Impact History and Regolith Evolution on the Moon: Geochemistry and ages of glasses from the apollo 16 site. *J. Geophys. Res. Planets* **124**, 3167–3180 (2019).

41. O. Eugster, G. F. Herzog, K. Marti, M. W. Caffee, Irradiation records, cosmic-ray exposure ages, and transfer times of meteorites, in *Meteorites and the Early Solar System II*, D. S. Lauretta, H. Y. McSween, Eds. (Univ. of Arizona Press, 2006), pp. 829.

42. K. H. Joy, I. A. Crawford, N. M. Curran, M. Zolensky, A. F. Fagan, D. A. Kring, The Moon: An archive of small body migration in the solar system. *Earth Moon Planets* **118**, 133–158 (2016).

43. O. Eugster, Cosmic-ray exposure ages of meteorites and lunar rocks and their significance. *Geochemistry* **63**, 3–30 (2003).

44. G. F. Herzog, M. W. Caffee, Cosmic-ray exposure ages of meteorites, in *Meteorites and Cosmochemical Processes*, A. M. Davis, Ed. (Elsevier, 2014), vol. 1, pp. 419–454.

45. F. Terfelt, B. Schmitz, Asteroid break-ups and meteorite delivery to Earth the past 500 million years. *Proc. Natl. Acad. Sci. U.S.A.* **118**, e2020977118 (2021).

46. M. J. Cintala, R. A. F. Grieve, Scaling impact melting and crater dimensions: Implications for the lunar cratering record. *Meteorit. Planet. Sci.* **33**, 889–912 (1998).

47. B. Schmitz, S. Boschi, A. Cronholm, P. R. Heck, S. Monechi, A. Montanari, F. Terfelt, Fragments of late eocene earth-impacting asteroids linked to disturbance of asteroid belt. *Earth Planet. Sci. Lett.* **425**, 77–83 (2015).

48. F. T. Kyte, A meteorite from the Cretaceous/Tertiary boundary. *Nature* **396**, 237–239 (1998).

49. D. Nesvorný, W. F. Bottke, S. Marchi, Dark primitive asteroids account for a large share of K/Pg-scale impacts on the Earth. *Icarus* **368**, 114621 (2021).

50. S. Desch, A. Jackson, J. Noviello, A. Anbar, The Chicxulub impactor: Comet or asteroid? *Astron. Geophys.* **62**, 3.34–33.37 (2021).

51. M. Schmieder, D. A. Kring, Earth's impact events through geologic time: A list of recommended ages for terrestrial impact structures and deposits. *Astrobiology* **20**, 91–141 (2020).

52. K. A. Farley, A. Montanari, E. M. Shoemaker, C. S. Shoemaker, Geochemical evidence for a comet shower in the late eocene. *Science* **280**, 1250–1253 (1998).

53. S. Boschi, B. Schmitz, P. R. Heck, A. Cronholm, C. Defouilloy, N. T. Kita, S. Monechi, A. Montanari, S. S. Rout, F. Terfelt, Late Eocene 3He and Ir anomalies associated with ordinary chondritic spinels. *Geochim. Cosmochim. Acta* **204**, 205–218 (2017).

54. D. D. Bogard, D. H. Garrison, M. Norman, E. R. D. Scott, K. Keil, ^{39}Ar-^{40}Ar age and petrology of Chico: Large-scale impact melting on the L chondrite parent body. *Geochim. Cosmochim. Acta* **59**, 1383–1399 (1995).

55. E. V. Korochantseva, M. Trieloeff, C. A. Lorenz, A. I. Buykin, M. A. Ivanova, W. H. Schwarz, J. Hopp, E. K. Jessberger, L-chondrite asteroid breakup tied to Ordovician meteorite shower by multiple isochron ^{40}Ar-^{39}Ar dating. *Meteorit. Planet. Sci.* **42**, 113–130 (2007).

56. S. Liao, M. H. Huyskens, Q.-Z. Yin, B. Schmitz, Absolute dating of the L-chondrite parent body breakup with high-precision U–Pb zircon geochronology from Ordovician limestone. *Earth Planet. Sci. Lett.* **547**, 116442 (2020).

57. F. Spoto, A. Milani, Z. Knežević, Asteroid family ages. *Icarus* **257**, 275–289 (2015).

58. P. W. Reiners, A. V. Turchyn, Extraterrestrial dust, the marine lithologic record, and global biogeochemical cycles. *Geology* **46**, 863–866 (2018).

59. H.-C. Tian, H. Wang, Y. Chen, W. Yang, Q. Zhou, C. Zhang, H.-L. Lin, C. Huang, S.-T. Wu, L.-H. Jia, L. Xu, D. Zhang, X.-G. Li, R. Chang, Y.-H. Yang, L.-W. Xie, D.-P. Zhang, G.-L. Zhang, S.-H. Yang, F.-Y. Wu, Non-KREEP origin for Chang'E-5 basalts in the Procellarum KREEP Terrane. *Nature* **600**, 59–63 (2021).

60. A. A. Nemchin, T. Long, B. L. Jolliff, Y. Wan, J. F. Snape, R. Zeigler, M. L. Grange, D. Liu, M. J. Whitehouse, N. E. Timms, F. Jourdan, Ages of lunar impact breccias: Limits for timing of the Imbrium impact. *Geochemistry* **81**, 125683 (2021).

61. J. D. Woodhead, J. M. Hergt, Pb-Isotope analyses of USGS reference materials. *Geostandards Newsletter* **24**, 33–38 (2000).

62. N Shimizu, S. R. Hart, Applications of the ion microprobe to geochemistry and cosmochemistry. *Annu. Rev. Earth Planet. Sci.* **10**, 483–526 (1982).

63. D. Weis, B. Kieffer, C. Maerschalk, W. Pretorius, J. Barling, High-precision Pb-Sr-Nd-Hf isotopic characterization of USGS BHVO-1 and BHVO-2 reference materials. *Geochem. Geophys. Geosyst.* **6**, Q02002 (2005).

64. M. Elburg, P. Vroon, B. van der Wagt, A. Tchalikian, Sr and Pb isotopic composition of five USGS glasses (BHVO-2G, BIR-1G, BCR-2G, TB-1G, NKT-1G). *Chem. Geol.* **223**, 196–207 (2005).

65. J. F. Snape, A. A. Nemchin, J. J. Bellucci, M. J. Whitehouse, R. Tartèse, J. J. Barnes, M. Anand, I. A. Crawford, K. H. Joy, Lunar basalt chronology, mantle differentiation and implications for determining the age of the Moon. *Earth Planet. Sci. Lett.* **451**, 149–158 (2016).

66. L. T. Silver, Lead volatilization and volatile transfer processes on the moon. *Lunar Planet. Sci. Conf.* **3**, 701–703 (1972).

67. L. T. Silver, Volatile lead components in lunar regolith at the apollo mare sites. *Lunar Planet. Sci. Conf.* **6**, 738–740 (1975).

68. A. M. Gaffney, L. E. Borg, Y. Asmerom, The origin of geochemical diversity of lunar mantle sources inferred from the combined U–Pb, Rb–Sr, and Sm–Nd isotope systematics of mare basalt 10017. *Geochim. Cosmochim. Acta* **71**, 3656–3671 (2007).

69. M. Tatsumoto, Age of the moon: An isotopic study of U-Th-Pb systematics of apollo 11 lunar samples-II. *Geochim. Cosmochim. Acta* **1**, 1595–1612 (1970).

70. T. Andersen, Correction of common lead in U–Pb analyses that do not report 204Pb. *Chem. Geol.* **192**, 59–79 (2002).

71. K. R. Ludwig, On the treatment of concordant uranium-lead ages. *Geochim. Cosmochim. Acta* **62**, 665–676 (1998).

72. W. Benz, A. G. W. Cameron, H. J. Melosh, The origin of the moon and the single-impact hypothesis III. *Icarus* **81**, 113–131 (1989).

73. E. Pierazzo, N. A. Artemieva, B. A. Ivanov, T. Kenkmann, F. Hörz, A. Deutsch, Starting conditions for hydrothermal systems underneath Martian craters: Hydrocode modeling, in *Large Meteorite Impacts III* (Geological Society of America, 2005), vol. 384, pp. 443–457.

74. A. Rajšić, K. Miljković, N. Wójcicka, G. S. Collins, K. Onodera, T. Kawamura, P. Lognonné, M. A. Wieczorek, I. J. Daubar, Numerical simulations of the Apollo S-IVB artificial impacts on the moon. *Earth Space Sci.* **8**, e2021EA001887 (2021).
